# Probing Hydrogen-Bonding Preferences and Methyl Internal Rotation in Sotolon and Sotolon-(H_2_O)_1,2_

**DOI:** 10.3390/ijms26125806

**Published:** 2025-06-17

**Authors:** Andrés Verde, Juan Carlos López, Susana Blanco

**Affiliations:** Departamento de Química Física y Química Inorgánica, Facultad de Ciencias, IU CINQUIMA, Universidad de Valladolid, 47011 Valladolid, Spain; andres.verde@uva.es (A.V.); juancarlos.lopez@uva.es (J.C.L.)

**Keywords:** hydrogen-bonding, rotational spectroscopy, large-amplitude vibrations, flavor compound, supersonic jets, microsolvation

## Abstract

Sotolon is a chiral furanone derivative featuring three distinct oxygen atoms at carbonyl, hydroxyl, and cyclic ether groups that can serve as hydrogen-bond acceptor sites, making it an ideal model system for probing water’s preferential interactions with competing functional groups. In this study, the rotational spectrum of sotolon and its microsolvated complexes, representing the early stages of hydration, was investigated using chirped-pulse Fourier transform microwave (CP-FTMW) spectroscopy. The conformational landscape of sotolon is dominated by a single conformer stabilized by an intramolecular O–H···O=C hydrogen bond. During hydration, water molecules disrupt this interaction by forming closed hydrogen-bonded cycles, resulting in mono- and dihydrated complexes. High-level theoretical calculations underscore the central role of electrostatic interactions in stabilizing these hydrated structures. Furthermore, *A*/*E* splittings observed in the rotational spectrum, arising from the internal rotation of one of sotolon’s methyl groups, provide insight into how hydration modulates the methyl internal rotation barrier.

## 1. Introduction

Hydrogen bonding (HB) is one of the most extensively studied types of non-covalent interactions, owing to its ubiquity in both chemistry and biology. Its significance is particularly evident in processes such as protein folding, structural stabilization of proteins, and molecular recognition, among many others [1,2,3]. These weak interactions occur predominantly in the solid and liquid phases and play a crucial role in the crystal packing of organic compounds, as well as in the three-dimensional architecture of biological macromolecules [4,5]. Due to their dual role as proton donors and acceptors, water molecules form extensive hydrogen-bonding networks that predominantly govern their interactions with solutes.

The investigation of small clusters involving water molecules, commonly referred to as microsolvation, is of considerable biological and chemical interest, as it enables a deeper understanding of the strength and nature of HBs and other intermolecular interactions. Moreover, the study of microsolvates is particularly valuable for examining the stepwise hydration of organic molecules. This approach allows researchers to determine the conditions under which droplet-like aggregation, where water molecules form self-associated networks resembling those in pure water clusters, is favored over the wetting pathway, where significant deviations from the behavior of pure water clusters are observed [6]. These molecular clusters can be generated and isolated in a controlled manner within the cold environment of supersonic jet expansions and subsequently detected using high-resolution spectroscopic techniques.

Rotational spectroscopy has been widely employed to investigate clusters containing increasing numbers of water molecules, thereby revealing the remarkable adaptability of water to various solutes [7,8,9,10]. Owing to the high structural resolution of this technique, several notable features have been observed in different hydrates, particularly those arising from cooperative effects, as exemplified by formamide–(H_2_O)_n_ complexes [11]. Given the significance of chirality in biological systems, microsolvated clusters of chiral molecules have also been characterized using this method [12]. In this context, recent advances in rotational spectroscopy, such as three-wave mixing and chiral tagging, have enabled the detailed investigation of chiral molecules in the gas phase with unprecedent sensitivity and specificity [13,14]. For example, the combined application of the enantioselective synthesis and analysis of isotopically chiral molecules through chiral tag rotational spectroscopy enables precise determination of absolute configuration and enantiomeric excess without reference samples, offering a robust framework for studying non-covalent interactions in gas-phase systems [14].

In addition, rotational spectroscopy enables the analysis of intramolecular dynamics in the detected systems, including methyl internal rotation. This motion induces characteristic splittings in the rotational spectrum, known as *A*/*E* splittings, arising from the interaction between overall molecular rotation and the torsional motion of the methyl group. The magnitude and pattern of these splittings provide direct insight into the internal rotation potential and allow for the accurate determination of the methyl torsional barrier [12,15]. Studies of methyl carbamate complexes with up to three water molecules reveal an increase in the internal rotation barrier with increasing hydration [15], a trend also observed for methyl lactate and its microsolvates in the gas phase [16]. Conversely, in the case of 3-methylcatechol, the addition of up to five water molecules has only a minimal effect on the methyl internal rotation barrier [6,17]. Interestingly, in some systems, such as 4-methylthiazole, complexation with a single water molecule has been found to slightly decrease the barrier height compared to the monomer [18].

**Figure 1:**
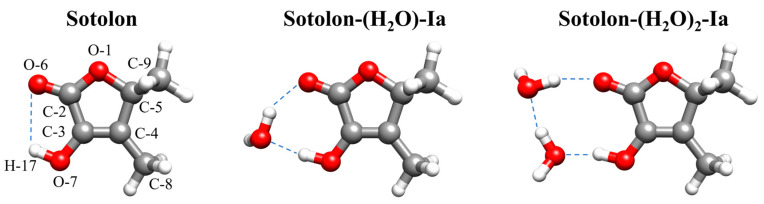
The detected conformers of sotolon, sotolon-(H_2_O), and sotolon-(H_2_O)_2_, which are also the most stable conformers of each system.

To investigate the preferential binding of water to distinct functional groups and to gain deeper insight into the influence of microsolvation on methyl internal rotation, sotolon was selected as an exemplary model system. Sotolon (3-hydroxy-4,5-dimethylfuran-2(5H)-one) is a lactone derivative of 2-furanone, characterized by three distinct oxygen atoms corresponding to cyclic ether, carbonyl, and hydroxyl functionalities (see Figure 1). This chiral furanone is recognized as a key flavor constituent in coffee, raw cane sugar, and dry white wine, among other matrices [19,20]. Its aromatic profile is exploited industrially due to its notable organoleptic properties and exceptionally low odor thresholds [21,22]. The *S*-enantiomer of sotolon, which exhibits a substantially lower perception threshold, predominantly contributes to the characteristic aroma of prematurely aged white wines [23]. Given its chirality, sotolon has been the subject of several experimental investigations employing vibrational circular dichroism and optical rotation techniques [22,24]. Furthermore, theoretical studies have demonstrated that sotolon possesses high activation barriers for enol/keto tautomerism processes [24]. A detailed elucidation of the molecular structure of sotolon and its interactions with other species is essential for understanding its specific binding mechanisms with olfactory receptors. To date, rotational spectroscopy has not been applied to the study of sotolon. Nonetheless, structural characterizations of analogous compounds, such as 2-acetylfuran and 2-methyltetrahydrofuran-3-one, have been reported [25,26]. Notably, for 2-acetylfuran, two conformers have been identified, both exhibiting methyl internal rotation with experimentally determined *V*_3_ barriers of 320 cm^−1^ and 230 cm^−1^, respectively [25]. The effect of microsolvation on the *V*_3_ barriers has been previously reported for molecules such as 4-methylthiazole [18], methyl carbamate [15], methyl lactate [16] and 3-methyl catechol [6]. Such literature data provide a basis to contextualize and complement the findings reported here.

In this study, we present a combined investigation using high-resolution rotational spectroscopy and quantum chemical calculations to elucidate the molecular structure of sotolon and its mono- and dihydrate complexes. The nature and strength of the potential hydrogen bonds are assessed through comprehensive molecular interaction analyses. Due to the presence of methyl groups, effects arising from internal rotation were observed in all detected species. The intramolecular dynamics of these methyl groups and the impact of microsolvation on their internal rotation barriers are also discussed. Thus, the novelty of this work lies in the accurate experimental and computational characterization of water binding preferences to an organic molecule presenting different functional groups, as well as in the analysis of how microsolvation influences the *V*_3_ barrier of a methyl group. Owing to the presence of a C_3_ chiral center, sotolon exists as both *R* and *S* enantiomers; however, within the scope of this investigation, both enantiomers exhibit identical spectroscopic properties. To avoid ambiguity, the *S*-enantiomer is used consistently throughout the text and figures.

## 2. Results and Discussion

### 2.1. Conformational Panorama

The conformational landscape of sotolon is dominated by a conformer stabilized by an intramolecular HB between the hydroxyl group and the carbonyl oxygen atom. A second conformer was predicted to lie 2030 cm^−1^ higher in energy according to the B3LYP-D3/6-311G++(d,p) level calculations (see Figure 1 and Figure S1 and Table S1).

**Figure 2:**
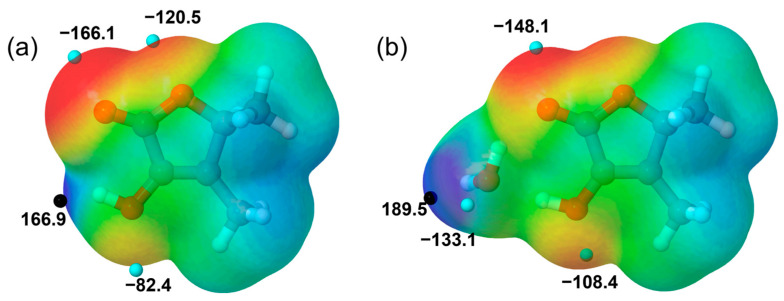
Molecular electrostatic potential of (**a**) sotolon and (**b**) sotolon-(H_2_O) most stable conformers on the 0.001 a.u. electron density isosurfaces. Blue and red colors show regions with positive and negative values, respectively. Maxima and minima with values exceeding ±80 kJ mol^−1^ are shown in black and light blue spheres.

Prior to examining the conformational panorama of the potential microsolvated complexes of sotolon, it is valuable to investigate the properties of the isolated most stable conformer. The molecular electrostatic potential surface (MEP) reveals that, due to the presence of different functional groups, one local maximum and several minima appear (see Figure 2a). The electrostatic potential maximum corresponds to the region of low electron density surrounding the hydroxyl hydrogen atom. In contrast, the more negative regions, indicative of high electron density, are localized around the oxygen atoms of the three distinct functional groups present in sotolon, each one with a different value.

In accordance with the MEP topology, the most stable monohydrates are associated with the maxima of 166.9 kJ mol^−1^. These conformers, herein referred to as sotolon-H_2_O Ia and Ib, are stabilized by the formation of a strong HB from the sotolon hydroxyl group to the oxygen atom of the water molecule (see Figure 1 and Figure S2). Furthermore, water closes a cycle with the solute through a second HB from water to the carbonyl group. Thus, water disrupts the intramolecular HB stabilizing sotolon. Both forms are practically isoenergetic and differ in the side of the ring plane to which the non-bonded water hydrogen atom is pointing (see Table S2 and Figure S2). Despite almost equal energies, these two conformers are non-equivalent due to the lack of symmetry in sotolon. The different orientation of water causes the *µ*_c_ component of the electric dipole moment to have opposite signs in both conformers. The predicted interconversion barrier between both complexes is 270 cm^−1^ (see Figure S4). The most stable monohydrate, lying 2131 cm^−1^ above, involves the water molecule interacting with the cyclic ether group and the nearest methyl group. In the remaining higher-energy conformers, the water molecule interacts either with two oxygen atoms simultaneously or with other functional groups, but these interactions are less favorable for stabilizing the monohydrated complex (see Table S2 and Figure S2). Monohydrates of the sotolon-2 conformer were not explored, as its high energy makes it unlikely to be populated in the molecular jet and therefore not detectable under the experimental conditions.

The MEP topology of sotolon-H_2_O also shows different maxima and minima as occurs for the monomer (see Figure 2b). Accordingly, the conformational landscape of the dihydrates is similarly dominated by a group of close-in-energy conformers, in which a water dimer forms a cycle with the hydroxyl and ketone groups through the formation of three HBs (see Figure 1). Variations in the arrangement of the two non-bonding hydrogen atoms of water result in four conformers. These forms are herein referred to as sotolon-(H_2_O)_2_ Ia, Ib, Ic, and Id. The next most stable dihydrate is predicted to lie 976 cm^−1^ higher in energy (see Table S3 and Figure S3).

For all conformers of the three systems studied, the MP2/6-311++G(d,p) level of theory confirms the significant stabilization of the lowest-energy conformers in each case predicted by DFT calculations. Moreover, the rotational constants predicted by both methods are in close agreement (see Tables S4–S6 for MP2 results).

### 2.2. Rotational Spectrum

**Figure 3:**
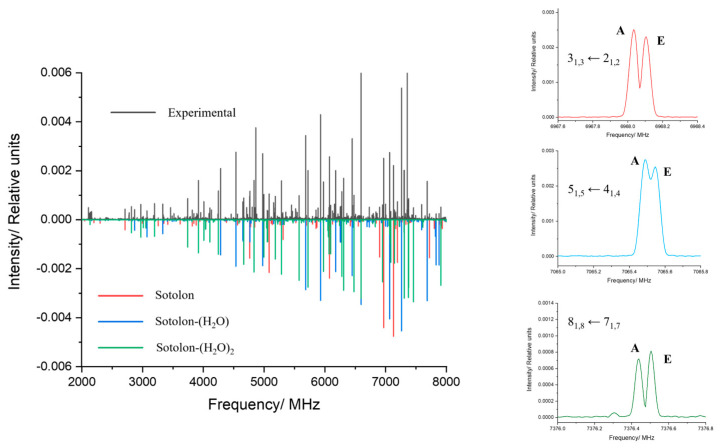
2–8 GHz experimental spectrum of sotolon in black. The spectrum calculated from the experimentally determined rotational parameters for sotolon is shown in red, for sotolon-(H_2_O) in blue and for sotolon-(H_2_O)_2_ in green. Example transitions with CH_3_ internal rotation characteristic tunneling splittings for the three detected systems are also shown.

The 2–8 GHz CP-FTMW spectrum of the Ne-sotolon-water mixture is shown in Figure 3. It shows sets of strong *a*-type and moderate *b*-type rotational transitions that were easily identified from the predicted rotational transitions of the most stable sotolon conformer. Each rotational transition splits into a doublet attributable to the *A* and *E* states of the internal rotation motion of a methyl group (see excerpts in Figure 3) with a reasonable low internal rotation barrier. The S/N observed do not allow the observation of the spectra of any heavy-atom isotopologue in natural abundance.

The most stable monohydrated conformers are predicted to be close to a prolate rotor with a value of the Ray asymmetry parameter [27] of −0.67 with a dominant *µ*_a_ dipole moment component but with reasonably high values of *µ*_b_ and *µ*_c_. The identification of the *a*-type spectrum of this species in the experimental spectrum was not difficult using the predicted rotational constants. Based on the initial set of observed rotational constants, the assignment of the *b*-type spectrum was straightforward. However, no evidence of *c*-type transitions was found. As in the case of the monomer, each transition appears as a doublet, attributed to *A/E* splittings (see Figure 3). The two low energy predicted conformers, which differ in the orientation of the non-bonded hydrogen atom of the water molecule, exhibit similar rotational constants that would result in a closely spaced spectrum. The potential energy function (Figure S4) predicts an interconversion barrier of 270 cm^−1^, Given the non-equivalence of the hydrate conformers, one might expect conformational relaxation from the higher-energy form to the global minimum during the supersonic expansion. But this is not compatible with the fact that no *c*-type spectrum has been observed. The calculated barrier height and the fact that the interconversion pathway essentially involves the rotation of H_2_O around the hydrogen bonded O-H bond that are hydrogen-bonded to sotolon, with a small vibrational mass, suggest that the ground vibrational state lies above the interconversion barrier. As a result, the vibrational wavefunction samples a wide range of rotation angles, and the expectation value relevant to the observed spectrum corresponds to an almost coplanar arrangement of the water molecule and sotolon ring, leading to a *µ*_c_ close to zero. As a result, only the spectrum of a single conformer with *a*- and *b*-type rotational transitions is observed. In consequence, the split lines were also fitted using the appropriate Hamiltonian for internal rotation.

When removing the measured transitions belonging to the sotolon hydrate, a survey of the spectrum shows the typical patterns corresponding to the *a*-type spectrum of a prolate rotor. Observed at full resolution, the new lines also show splittings attributable to the *A* and *E* states of a methyl rotor. The preliminary set of rotational constants derived from the observed spectrum supports its assignment to the most stable dihydrate, as evidenced by the close agreement between experimental and theoretical values.

The observation of only *a*-type transitions is consistent with the predicted large *µ_a_* dipole moment component and the comparatively small values of *µ_b_* and *µ_c_* (see Table S3), as calculated at the DFT level.

For each detected form, two independent fits for the *A* and *E* states were performed to the *A*-semirigid rotor Hamiltonian of Watson [28] in the I^r^ representation using Pickett’s SPFIT program [29]. In the analysis of the *E* states, the effective Hamiltonian was complemented with an additional term (${\hat{H}}_{int}$), which is defined as

(1) $${\hat{H}}_{int}=D_{a}\cdot {\hat{P}}_{a}+D_{b}\cdot {\hat{P}}_{b}+D_{c}\cdot {\hat{P}}_{c}$$

The results for the three observed rotamers are collected in Table 1. According to a perturbational treatment of the methyl group internal rotation problem [30,31] the differences between the rotational constants of the *A* and *E* states and the parameters *D_a_*, *D_b_* and *D_c_* can be written as

(2) $$∆A=A_{A}-A_{E}=F\rho_{a}^{2}\left[W_{A}^{\left(2\right)}-W_{E}^{\left(2\right)}\right]\;∆B=B_{A}-B_{E}=F\rho_{b}^{2}\left[W_{A}^{\left(2\right)}-W_{E}^{\left(2\right)}\right]\;∆C=C_{A}-C_{E}=F\rho_{c}^{2}\left[W_{A}^{\left(2\right)}-W_{E}^{\left(2\right)}\right]$$

(3) $$D_{a}=F\rho_{a}W_{E}^{\left(1\right)},\;D_{b}=F\rho_{b}W_{E}^{\left(1\right)}\mathrm{and}\;D_{c}=F\rho_{c}W_{E}^{\left(1\right)}$$

where *F* is the reduced rotational constant, related to the methyl group inertial moment *I*_α_ by

(4) $$F=\frac{\hbar^{2}}{2I_{\alpha }r}$$

(5) $$r=1-\sum_{g}\rho_{g}\lambda_{g}\;\;\left(g=a,b,c\right)$$

where *λ*_g_ (*g = a*,*b*,*c*) are the direction cosines giving the orientation of the internal rotation axis in the principal inertial axis system, *ρ*_g_ are the components of the vector *ρ*, defined in terms of the internal rotor moment of inertia *I*_α_, and the molecular moments of inertia *I*_g_ as

(6) $$\rho_{g}=\frac{\lambda_{g}I_{\alpha }}{I_{g}}$$

*W*_E_^(1)^ is the first order perturbation coefficient for the *E* state, whereas *W*_A_^(2)^ and *W*_E_^(2)^ are the corresponding second order perturbation coefficients for the *A* and *E* states, respectively. All these parameters can be calculated from the molecular structure. Those corresponding to the methyl groups are given for the detected species in Table S8. The perturbation coefficients can be taken from the tables of Herschbach [32] where they are given as a function of the reduced barrier:

(7) $$s=4V_{3}/9F$$

In this way, it is possible to relate the values of the rotational constants or the parameters *D*_a_, *D*_b_, and *D*_c_ with the reduced barrier *s* to estimate the value of the barrier *V*_3_.

For the three detected species, *D*_a_ and *D*_b_ were determined for the *E* state, whereas *D*_c_ has to be fixed to zero. The non-determination of *D*_c_ is in good agreement with values of *ρ*_c_ close to zero. Furthermore, the determined values are only consistent with the values of the *ρ* for the methyl group linked to the sp^2^ carbon atom in position 5 (see Figure 1 and Table S8). From the *D*_a_ and *D*_b_ parameters, the values estimated are s = 22–23 and values of V_3_ = 260–280 cm^−1^. Since the differences between the C rotational constants are very small, only the differences between the rotational constants A and B have been used to estimate values of s = 29–33 giving barriers V_3_ = 350–400 cm^−1^.

From those estimations, we have proceeded to fit both *A* and *E* states of each species simultaneously using the XIAM program [33] in order to obtain more accurate *V*_3_ barrier values. Initial values of methyl top inertial moment *I*_α_, the reduced rotational constant *F*, and the geometrical parameters describing the orientation of the methyl top were calculated from the available structures (see Table S8). This type of fit requires a preliminary calibration step in order to determine which parameters could be reliably fitted. The final fits determined using both SPFIT and XIAM packages (http://info.ifpan.edu.pl/~kisiel/prospe.htm#table_of_programs, accessed on 25 May 2025) are shown in Table 1. The barriers obtained from the XIAM fit seem to be in good agreement with the barriers estimated from the perturbation treatment using the differences between A and B rotational constants (Equation (3)). All the observed frequencies that have been used for both SPFIT and XIAM fits are given in Tables S12–S20.

### 2.3. Molecular Structure and Molecular Interactions

Considering the good agreement between the experimentally determined rotational constants and those calculated using DFT methods (B3LYP-D3/6-311++G(d,p)), we can assume that the predicted structures accurately reflect the experimental geometries. From the experimental data shown in Table 1, it can be observed that the rotational constant A remains similar for both the monomer and the monohydrate, suggesting that the water molecule is located near the *a*-inertia axis of sotolon. Additionally, the values of the planar moment *P_b_*, which quantifies the mass distribution out of the *ac* plane, are also relatively similar for both species, indicating that water lies close to the *ac* plane. Furthermore, the *Pc* values for the monomer, monohydrate, and dihydrate are all very close, placing water molecules near the *ab* plane of the molecule, which is nearly coincident with the plane of the sotolon ring.

It is well-established that a high HB strength is associated with short donor–acceptor distances and specific angular orientations. For instance, in the case of carbonyl functional groups, optimal C=O···H_w_ angles should approach 120°, as a consequence of the position of oxygen lone pairs. Consequently, an estimation of the HBs can be made using the DFT geometries (see Tables S9–S11), as these structures can be taken as a reasonably good prediction due to their close alignment with the experimental rotational parameters. The intramolecular interaction that stabilizes sotolon is *ca*. 2.413 Å and thus, it can be classified as a weak HB [2]. As previously mentioned, water disrupts this interaction in the monohydrate. When water acts as a HB acceptor, the O_7_H_17_···H_w_ distance is 1.733 Å and the angle formed is 173.96°, indicating nearly optimal linearity of the interaction. On the other hand, when water acts as a proton donor, the C_2_=O_6_···H_w_ is 1.854 Å and the angle is 121.14°. The analysis of the dihydrate reveals σ-bond cooperative effects. The O···O distance between water molecules is 2.721 Å, which is notably shorter than the experimental 2.98(4) Å of the isolated water dimer [34]. Furthermore, the O_7_H_17_···H_w_ and C_2_=O_6_···H_w_ distances are in this case predicted to be 1.711 Å and 1.812 Å, respectively. Nevertheless, the direccionability of the HBs is less favorable, exhibiting values of 164.65° and 159.14°, which can be attributed to the restricted spatial availability between the hydroxyl and the carbonyl functional groups of sotolon that does not allow a better disposition for the water dimer.

**Figure 4:**
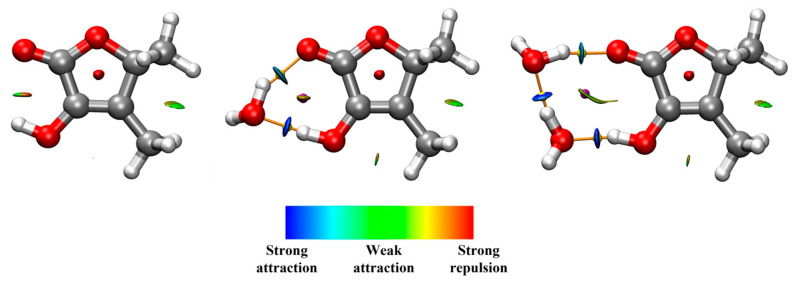
NCI and QTAIM combined analysis for sotolon, sotolon-H_2_O and sotolon-(H_2_O)_2_. QTAIM analysis shows (3, −1) bond critical points in yellow, (3, +1) ring critical points in purple and bond paths in orange. NCI plot maps the location and strength of molecular interactions with colored isosurfaces ranging from blue (strong attraction) to red (strong repulsion) according to the product of the sign of the second eigenvalue of the Hessian and electron density.

In order to gain more insights into the strength of the molecular interactions presented in the systems, NCI and Bader’s QTAIM analysis have been carried out (see Figure 4). Although the NCI plot shows a weak attractive interaction between the hydroxyl group and the ketone’s oxygen for the monomer, QTAIM does not show the presence of a (3, −1) bond critical point (BCP) for this interaction. In fact, the O-H···O distance and the angular geometry of this intramolecular contact confirm its weakness.

In the monohydrate, water easily disrupts the weak intramolecular interaction and thus, the complex is strongly stabilized by the formation of a seven-membered *pseudo* ring through the establishment of two HBs between water molecules and sotolon. QTAIM analysis revealed the existence of the BCPs corresponding to two HBs and the formation of a cycle with a (3, +1) ring critical point (RCP). In order to obtain a more quantitative view of the strength of the interactions, we have employed the relationship between the interaction energy and the electron density *ρ(r)* at a BCP proposed by Emamian et al. [35]. The interaction energies calculated for the two HBs of this complex are −35.0 kJmol^−1^ for the HB in which the hydroxyl group is involved and −25.7 kJmol^−1^ for the HB established between water and ketone.

According to the dihydrate structure, the HBs established between water molecules and sotolon form a nine-membered *pseudo* ring. The interaction energy for the interaction between water and the hydroxyl group is −36.8 kJmol^−1^. The fact that this strength is higher than in the monohydrate is consistent with σ-bond cooperativity. The strength of the HB between water molecules is −32.3 kJmol^−1^, while the interaction energy for the HB between water and the ketone’s group has a value of −24.2 kJmol^−1^.

NBO theoretical calculations provide a quantitative point of view of the mentioned interactions. This analysis reflects the intramolecular interaction in the sotolon monomer predicting an small electron density transfer from the O_6_ lone pair to the O_7_-H_17_ antibonding σ^*^ orbital resulting in a second order E^(2)^ perturbation energy of 2.30 kJ mol^−1^. For the O-H-O_water_ interaction in sotolon-(H_2_O), the electron density transfer from the water oxygen lone to the hydroxyl O-H σ^*^ antibonding orbital gives a second order E^(2)^ energy of 85.20 kJ mol^−1^. On the other hand, the second order E^(2)^ energy for the O_water_-H···O=C HB interaction delocalizating the electrondensity of the carbonyl lone pair into the O-H σ^*^ orbital of water is 30.92 kJ mol^−1^. When evaluating the dihydrate, NBO shows an electron density transfer with E^(2)^ = 93.47 kJ mol^−1^ for the HB between the hydroxyl group and water, E^(2)^ = 68.58 kJ mol^−1^ for the HB interaction between water molecules and E^(2)^ = 5.94 kJ mol^−1^ for the HB interaction between water and the carbonyl oxygen.

BSSE corrected dissociation energies of the detected systems calculated using the counterpoise method [36] are shown in Table 2. The positive value of the interaction energy represents the equilibrium dissociation energy *D_e_*. Table 2 also shows the average energy per HB, that has been calculated as *D_e_/n_HB_*, where n_HB_ is the number of HB present in the complex. Comparison between the *D_e_/n_HB_* value for the mono- and dihydrate show an increasing average energy per bond energy with the addition of the second water molecule, an indication of σ-bond cooperativity. The values found are also in agreement with the average of the HBs’ energies estimated from the QTAIM results using the equation proposed by Emamiam et al.

The interaction energy can be decomposed into its constituent physical components, facilitating a more profound comprehension of the factors that play key roles in the intermolecular interactions. We have employed the sobEDAw strategy [37], designed for dispersion corrected DFT calculations, to understand the main forces at play in the interactions between sotolon and water (see Table 3). The results found are also consistent with those obtained using SAPT(0)/jun-cc-pVDZ (see Table S7). ΔE_int_, which is the interaction energy, is close to the complexation energy (−*D*_e_) value found form the BSSE calculation. As it was expected, the major contribution at play is of electrostatic nature, which comes from the donor–acceptor character of water molecules that interconnect two sotolon moieties through the formation of HBs. This predominance of electrostatic forces is also observed in the dihydrate, in which the total energy is higher due to the presence of the three HBs. 

**Figure 5:**
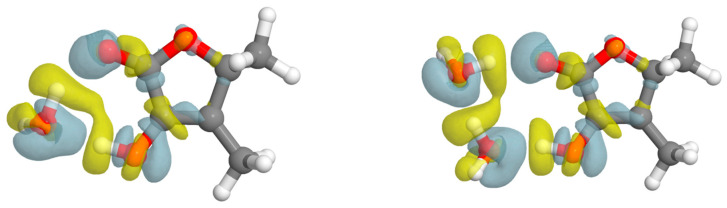
Electron density shifts in structures of sotolon-(H_2_O)_1,2_ calculated at B3LYP-D3/6-311++G(d,p) level. Blue and yellow colors represent regions with gain and loss of charge (±0.001 au), respectively.

The electron density shifts (EDS) [38] maps allow the visualizations of the changes in the electron density of a given complex upon complexation. For both microhydrates, the plot clearly shows the loss (yellow) and gain (blue) regions in the space of the functional groups participating in the HB interactions (see Figure 5). It is worth noting that even the oxygen ester of sotolon, which is not directly involved in establishing HBs with water, suffers some redistribution of charge. Thus, it is to some extent affected by the interactions of sotolon with water. This phenomenon can also be observed in the non-bonded hydrogen atoms of water.

### 2.4. Methyl Internal Rotation

The observed splittings in the spectrum have been attributed to the methyl internal rotation of one of the two methyl groups of sotolon. The *V*_3_ barrier for the methyl internal rotation of the methyl group linked to the sp^3^ hybridation C_5_, has been calculated to be 1214 cm^−1^ at B3LYP-D3/6-311++G(d,p) level of theory. This value is predicted to remain relatively constant with the addition of water, leading to barriers of 1212 cm^−1^ and 1196 cm^−1^ for the mono- and dihydrate, respectively. These predicted interconversion barriers are high enough to quench the observation of *A/E* splittings in the ground vibrational state rotational spectrum.

Conversely, the DFT values of the *V*_3_ barriers calculated for the C_8_ methyl group linked to sp^2^-hybridized C_4_ are 326 cm^−1^, 294 cm^−1^ and 298 cm^−1^ for the monomer, the monohydrate and the dihydrate, respectively. The experimental barriers determined from analysis of the spectra using XIAM program are 372.38(43) cm^−1^, 348.78(24) cm^−1^ and 345.808(74) cm^−1^ respectively. The identification of these barriers as belonging to this methyl group is also supported by the geometrical parameters associated with this vibration determined experimentally (see Table S8). Note that the theoretical barrier values are lower than the experimental ones; however, they effectively replicate the trends of the methyl internal rotation barrier upon successive addition of water to sotolon in the mono and dihydrate. The addition of the first water molecule has the effect of reducing the value of the interconversion barrier by approximately 30 cm^−1^. However, the addition of a second water molecule does not result in a significant alteration in the value of the barrier. The comparison between the theoretical and experimental interconversion barriers is shown in Table 4. The observed effect of the water addition could be a consequence of the redistribution of charge resulting from the formation of HBs formed between water and sotolon. This behavior is analogous to that observed for 4-methylthiazole [18] but contrary to those of methyl carbamate [15] or methyl lactate [16]. The fact that the addition of the second molecule does not affect the value with respect to the monohydrate is also consistent with the effect observed in 3-methylcatechol [6,17].

## 3. Materials and Methods

### 3.1. Experimental Details

The rotational spectrum of sotolon was recorded in the frequency range of 2 to 8 GHz with a CP-FTMW spectrometer described elsewhere [39,40]. A commercial sample of sotolon was placed in a heatable reservoir at the nozzle and maintained at 150 °C. The resulting sotolon vapor was seeded in a neon stream at a stagnation pressure of 2 bar. A supersonic jet was generated by the expansion of the gas mixture through a small diameter nozzle into a high-vacuum chamber. Microwave excitation was achieved using multifrequency chirped pulses of 4 μs duration, generated by an arbitrary waveform generator (AWG) and amplified to 200 W with a traveling wave tube (TWT) amplifier. The induced molecular polarization was broadcast by a horn antenna oriented perpendicular to the expansion axis. The subsequent free induction decay (FID) signal was detected by a second horn antenna, digitized using a high-speed oscilloscope, and Fourier transformed to yield the frequency-domain spectrum.

Each expansion was probed with eight acquisition cycles, and the resulting spectra were averaged. The experiment was conducted at a repetition rate of 5 Hz, providing sufficient time for the vacuum chamber to recover between cycles.

For the sotolon–water complexes, water vapor was introduced by placing a small reservoir of liquid water in the neon gas line upstream of the nozzle, enabling co-expansion and efficient microsolvation.

### 3.2. Theoretical Methods

The conformational preferences of sotolon, sotolon-(H_2_O) and sotolon-(H_2_O)_2_ systems were investigated with the aid of Conformer-Rotamer Ensemble Sampling (CREST) tool (https://crest-lab.github.io/crest-docs/, accessed on 25 May 2025) [41,42]. All the predicted conformers and complexes were further optimized employing the B3LYP hybrid density functional [43]. with the D3 Grime’s dispersion correction term [44] and the Pople’s 6-311++G(d,p) basis set [45]. This level of theory has been shown to provide reliable predictions of rotational constants for both isolated molecules and molecular clusters, making it particularly suitable for the present investigation [46,47]. Harmonic frequency calculations were also carried out to ensure that all the conformers are indeed minima in the potential energy surface. Furthermore, ab initio MP2 [48] optimizations were also performed for all the potential conformers and complexes. The internal rotation barrier (*V*_3_) of both methyl groups was calculated for the detected species using the DFT method. All the calculations were performed using GAUSSIAN16 software package [49]. The maxima and minima in the molecular electrostatic potentials have been calculated on the 0.001 a.u. electron density isosurface with the Multiwfn program (http://sobereva.com/multiwfn/, accessed on 25 May 2025) [50].

Non-Covalent Interactions (NCI) [51], Bader’s Quantum theory of Atoms in Molecules (QTAIM) [52], and Natural Bond Orbitals (NBO) [53] analysis were conducted to provide insights about the nature and strength of the molecular interactions. B3LYP-D3/6-311++G(d,p) calculations were also performed to estimate the dissociation energies of the detected complexes using the counterpoise method to correct the basis set superposition error (BSSE) [36]. Energy decomposition analyses were performed using symmetry adapted perturbation theory (SAPT) calculations [54,55] and alternatively following the sobEDAw method [37] were carried out to obtain information about the nature of the intermolecular interactions that stabilize the complexes using PSI4 (https://psicode.org/psi4manual/master/index.html, accessed on 25 May 2025) [56] and Multiwfn [50] programs, respectively.

Moreover, the electron density shift [38] upon complexation was evaluated as the difference in the electron density of the complex and that of the monomers in the geometry of the complex.

## 4. Conclusions

In summary, the results presented here highlight the novelty of combining high-resolution rotational spectroscopy and quantum chemical methods to investigate how water selectively interacts with different functional groups of a given organic molecule. In particular, the conformational preferences of sotolon and sotolon-(H_2_O)_1–2_ have been unveiled. The broadband rotational spectrum reveals that the most stable conformer is stabilized by a weak O-H···O=C intramolecular interaction. The addition of water easily disrupts this interaction, with the water molecule(s) linking the hydroxyl and ketone moieties of sotolon through strong HBs, allowing the formation of a seven-membered *pseudo* ring in the monohydrate and a nine-membered *pseudo* ring in the dihydrate. Analysis of intermolecular interactions shows that electrostatic contributions are the most significant. They represent ~65% of the total attractive interactions in both microsolvates.

The observation of *A/E* splittings in the rotational spectrum and their detailed analysis have allowed us to obtain new information about the internal dynamics of sotolon in the monomer and hydrates. Only the methyl group attached to the sp^2^-hybridized carbon atom exhibits an interconversion barrier low enough to display *A/E* splittings in the ground vibrational state rotational spectrum. The analysis of the experimental data has allowed us to determine the *V*_3_ internal rotation barrier for this methyl group in the detected forms. The addition of the first water molecule and the establishment of two HBs result in a slight decrease in the barrier with respect to the monomer. This might be attributed to the internal electronic change redistribution in sotolon upon complexation. However, the addition of a second water molecule has a clearly less pronounced effect on the value of the barrier. The investigation of large amplitude vibrations, such as methyl group internal rotation, in molecules and their complexes provides a powerful probe that reveals how complexation influences molecular properties that would otherwise remain undetected by standard analytical approaches.

Future experimental and theoretical investigations may reveal whether microsolvated clusters containing three or more water molecules continue to grow in a chain-like trend or as interaction between the monomer and the pure water cluster, whether such extended solvation induces any detectable structural changes in the chiral solute, and how the internal rotation barrier evolves upon further hydration. This work can also set a basis for future chiral tagging investigations.

## Figures and Tables

**Table 1:** Experimental rotational parameters obtained for sotolon and sotolon-(H_2_O)_1–2_ species using SPFIT and XIAM packages.

	Sotolon			Sotolon-(H_2_O)		
	SPFIT		XIAM	SPFIT		XIAM
Param. ^a^	*A*	*E*		*A*	*E*	
*A*/MHz	2232.14921(35) ^b^	2232.0820(17)	2232.09770(49)	2113.48296(59)	2113.43803(57)	2113.44992(56)
*B*/MHz	1683.79965(26)	1683.73308(68)	1683.76052(33)	890.95221(48)	890.91708(16)	890.92982(20)
*C*/MHz	1026.48763(23)	1026.48750(42)	1026.48327(28)	659.47245(45)	659.47015(14)	659.46969(19)
Δ*_J_*/kHz	-	-	-	0.051(10)	-	-
Δ*_JK_*/kHz	-	0.417(87)	-	-	-	-
Δ*_K_*/kHz	-	-	-	-	-	-
*D_a_*/MHz	-	−4.933(15)	-	-	−4.4371(23)	-
*D_b_*/MHz	-	−3.63(34)	-	-	−3.73(12)	-
*P_a_*/uÅ^2^	283.035549(95)	283.04151(25)	283.03747(12)	547.22591(45)	547.23588(17)	547.23277(31)
*P_b_*/uÅ^2^	209.302678(95)	209.29678(25)	209.30285(12)	219.11255(45)	219.10525(17)	219.10890(31)
*P_c_*/uÅ^2^	17.106514(95)	17.11242(25)	17.11157(12)	20.00889(45)	20.02128(17)	20.01629(31)
*s*			30.73			29.02
*V*_3_/cm^−1^	-	-	372.38(43)	-	-	348.78(24)
*F*/GHz	-	-	161.45(derived)	-	-	160.15 (derived)
*F*_0_/GHz	-	-	159.521(93)	-	-	158.973(54)
*I_α_*/uÅ^2^	-	-	3.1681(18)	-	-	3.1790(11)
*ρ*/deg	-	-	0.012(calculated)	-	-	0.0080(calculated)
*β*/deg	-	-	0.7390(20)	-	-	2.48(calculated)
*γ*	-	-	0.0146(16)	-	-	3.107(23)
*µ_a_*/*D*	YES	YES	YES	YES	YES	YES
*µ_b_*/*D*	YES	YES	YES	YES	YES	YES
*µ_c_*/*D*	NO	NO	NO	NO	NO	NO
*N*	30	30	60	48	39	82
*σ*/kHz	3.6	5.8	6.5	6.9	4.3	8.8
		**Sotolon-(H_2_O)_2_**				
		SPFIT				XIAM
Param. ^a^		*A*		*E*		
*A*/MHz		1680.068(25)		1680.038(39)		1680.094(21)
*B*/MHz		565.40988(53)		565.40074(58)		565.40482(43)
*C*/MHz		438.63777(62)		438.63742(65)		438.63699(49)
Δ*_J_*/kHz		0.0106(47)		0.0465(53)		0.0284(39)
Δ*_JK_*/kHz		0.177(35)		-		0.136(25)
Δ*_K_*/kHz		-		2.23(67)		-
*D_a_*/MHz		-		−4.4339(41)		-
*D_b_*/MHz		-		−2.31(54)		-
*P_a_*/uÅ^2^		872.5875(36)		873.5925(48)		872.5949(29)
*P_b_*/uÅ^2^		279.5684(36)		279.5643(48)		279.5631(29)
*P_c_*/uÅ^2^		21.2403(36)		21.2498(48)		21.2410(29)
*s*						28.81
*V_3_*/cm^−1^						345.808(74)
*F*/GHz		-		-		159.93(derived)
*F*_0_/GHz		-		-		158.99(derived)
*I_α_*/uÅ^2^		-		-		3.18
*ρ*/deg		-		-		0.0068(calculated)
*β*/deg		-		-		0.44(calculated)
*γ*		-		-		0.054(calculated)
*µ_a_*/*D*		YES		YES		YES
*µ_b_*/*D*		NO		NO		NO
*µ_c_*/*D*		NO		NO		NO
*N*		33		31		68
*σ*/kHz		7.5		6.9		8.8

^a^ A, B and C are the rotational constants. Δ_J_, Δ_K_ and Δ_JK_ are the centrifugal distortion constants. D_a_ and D_b_ are coefficient terms related to the internal rotation (see Equation (1)). P_a_, P_b_ and P_c_ are the planar moments of inertia. s is the reduced barrier of the internal rotation. V_3_ is the barrier of the internal rotation. I_α_ is the moment of inertia of the internal rotation. F is the reduced internal rotation constant and F_0_ is directly related to the inverse moment of inertia of the methyl top, I_α_. ρ is the dimensionless module of the vector ρ essential to describe the coupling of internal and overall rotation. β is the angle between the principal inertial axis, a, and ρ. μ_α_ (α = a, b, or c) are the electric dipole. ^b^ Standard error in parenthesis in units of the last digit.

**Table 2:** Predicted equilibrium dissociation energies (*D_e_*) and average dissociation energies per HB (*D_e_/n_HB_*) for the detected complexes of sotolon calculated at B3LYP-D3/6-311++G(d,p) level of theory and employing the BSSE method.

	Sotolon-(H_2_O)	Sotolon-(H_2_O)_2_
*D_e_*/kJmol^−1^	56.74	100.00
*D_e_*/n_HB_/kJmol^−1^	28.37	33.33

**Table 3:** Energy decomposition (kJ mol^−1^) for sotolon complexes obtained from the sobEDAw analysis performed at B3LY-D3(BJ)/def2TZVP level of theory. ΔE_eles_ represent the electrostatic contribution, ΔE_ex-rep_ the exchange-repulsion, ΔE_orb_ the orbital energy and ΔE_disp_ the dispersion forces.

	ΔE_eles_	ΔE_ex-rep_	ΔE_orb_	ΔE_disp_	ΔE_int_
Sotolon-(H_2_O)	−107.6	136.6	−55.4	−31.5	−57.9
Sotolon-(H_2_O)_2_	−161.5	202.4	−90.6	−50.7	−100.4

**Table 4:** Internal rotation *V*_3_ barriers in cm^−1^ for the detected systems. DFT theoretical values were predicted with B3LYP-D3/6-311++G(d,p) method. The experimental values were determined using the XIAM program.

	DFT	Experiment
Sotolon	326	372.38(43)
Sotolon-(H_2_O)	294	348.78(24)
Sotolon-(H_2_O)_2_	298	345.808(74)

## Data Availability

The data that support the findings of this work are available in the Supplementary Material of this article.

## Supplementary Materials

The following supporting information can be downloaded at https://www.mdpi.com/article/10.3390/ijms26125806/s1.

## References

[1] Jeffrey G.A. (1997). An Introduction to Hydrogen Bonding.

[2] Desiraju G.R., Steiner T. (1999). The Weak Hydrogen Bond in Structural Chemistry and Biology.

[3] Martín-Fernández C., Montero-Campillo M.M., Alkorta I. (2024). Hydrogen Bonds Are Never of an “Anti-Electrostatic” Nature: A Brief Tour of a Misleading Nomenclature. J. Phys. Chem. Lett..

[4] Lehn J.-M. (2002). Toward Self-Organization and Complex Matter. Science.

[5] Busch S., Bruce C.D., Redfield C., Lorenz C.D., McLain S.E. (2013). Water Mediation Is Essential to Nucleation of β-Turn Formation in Peptide Folding Motifs. Angew. Chem. Int. Ed..

[6] Hazrah A.S., Insausti A., Ma J., Al-Jabiri M.H., Jäger W., Xu Y. (2023). Wetting vs. Droplet Aggregation: A Broadband Rotational Spectroscopic Study of 3-Methylcatechol⋅⋅⋅Water Clusters. Angew. Chem. Int. Ed..

[7] Burevschi E., Chrayteh M., Murugachandran S.I., Loru D., Dréan P., Sanz M.E. (2024). Water Arrangements upon Interaction with a Rigid Solute: Multiconfigurational Fenchone-(H_2_O)_4–7_ Hydrates. J. Am. Chem. Soc..

[8] Macario A., López J.C., Blanco S. (2024). Molecular Structure of Salicylic Acid and Its Hydrates: A Rotational Spectroscopy Study. Int. J. Mol. Sci..

[9] Steber A.L., Temelso B., Kisiel Z., Schnell M., Pérez C. (2023). Rotational Dive into the Water Clusters on a Simple Sugar Substrate. Proc. Natl. Acad. Sci. USA.

[10] Pérez C., López J.C., Blanco S., Schnell M. (2016). Water-Induced Structural Changes in Crown Ethers from Broadband Rotational Spectroscopy. J. Phys. Chem. Lett..

[11] Blanco S., Pinacho P., López J.C. (2017). Structure and Dynamics in Formamide–(H_2_O)_3_: A Water Pentamer Analogue. J. Phys. Chem. Lett..

[12] Krin A., Pérez C., Pinacho P., Quesada-Moreno M.M., López-González J.J., Avilés-Moreno J.R., Blanco S., López J.C., Schnell M. (2018). Structure Determination, Conformational Flexibility, Internal Dynamics, and Chiral Analysis of Pulegone and Its Complex with Water. Chem. A Eur. J..

[13] Domingos S.R., Pérez C., Marshall M.D., Leung H.O., Schnell M. (2020). Assessing the Performance of Rotational Spectroscopy in Chiral Analysis. Chem. Sci..

[14] Mills M.D., Sonstrom R.E., Vang Z.P., Neill J.L., Scolati H.N., West C.T., Pate B.H., Clark J.R. (2022). Enantioselective Synthesis of Enantioisotopomers with Quantitative Chiral Analysis by Chiral Tag Rotational Spectroscopy. Angew. Chem. Int. Ed..

[15] Pinacho P., López J.C., Kisiel Z., Blanco S. (2024). The Effect of Microsolvation on the Structure, Nuclear Quadrupole Coupling, and Internal Rotation: The Methyl Carbamate⋯(H_2_O)_1–3_ Complexes. J. Chem. Phys..

[16] Thomas J., Sukhorukov O., Jäger W., Xu Y. (2014). Direct Spectroscopic Detection of the Orientation of Free OH Groups in Methyl Lactate–(Water)_1,2_ Clusters: Hydration of a Chiral Hydroxy Ester. Angew. Chem. Int. Ed..

[17] Hazrah A.S., Al-Jabiri M.H., Jäger W. (2022). Structure and Conformations of 3-Methylcatechol: A Rotational Spectroscopic and Theoretical Study. J. Mol. Spectrosc..

[18] Cummings C.N., Kleiner I., Walker N.R. (2023). Noncovalent Interactions in the Molecular Geometries of 4-Methylthiazole···H_2_O and 5-Methylthiazole···H_2_O Revealed by Microwave Spectroscopy. J. Phys. Chem. A.

[19] Blank I., Sen A., Grosch W. (1992). Potent Odorants of the Roasted Powder and Brew of Arabica Coffee. Z. Lebensm. Unters. Forsch..

[20] Pons A., Lavigne V., Landais Y., Darriet P., Dubourdieu D. (2010). Identification of a Sotolon Pathway in Dry White Wines. J. Agric. Food Chem..

[21] Martin B., Etievant P.X., Le Quere J.L., Schlich P. (1992). More Clues about Sensory Impact of Sotolon in Some Flor Sherry Wines. J. Agric. Food Chem..

[22] Nakahashi A., Yaguchi Y., Miura N., Emura M., Monde K. (2011). A Vibrational Circular Dichroism Approach to the Determination of the Absolute Configurations of Flavorous 5-Substituted-2(5*H*)-Furanones. J. Nat. Prod..

[23] Pons A., Lavigne V., Landais Y., Darriet P., Dubourdieu D. (2008). Distribution and Organoleptic Impact of Sotolon Enantiomers in Dry White Wines. J. Agric. Food Chem..

[24] Yang Q., Liang M.-M., Wang H.-J., Zhao Q.-Q., Zhu H.-J., Liu L., Pittman C.U. (2017). Investigating Cyclic Sotolon, Maple Furanone and Their Dimers in Solution Using Optical Rotation, Electronic Circular Dichroism and Vibrational Circular Dichroism. Tetrahedron.

[25] Dindić C., Lüchow A., Vogt N., Demaison J., Nguyen H.V.L. (2021). Equilibrium Structure in the Presence of Methyl Internal Rotation: Microwave Spectroscopy and Quantum Chemistry Study of the Two Conformers of 2-Acetylfuran. J. Phys. Chem. A.

[26] Van V., Stahl W., Nguyen M.T., Nguyen H.V.L. (2020). The Smell of Coffee: The Carbon Atom Microwave Structure of Coffee Furanone Validated by Quantum Chemistry. Can. J. Phys..

[27] Ray B.S. (1932). Uber Die Eigenwerte Des Asymmetrischen Kreisels. Z. Phys..

[28] Watson J.K.G., During J.R. (1997). Aspects of Quartic and Sextic Centrifugal Effects on Rotational Energy Levels. Vibrational Spectra and Structure a Series of Advances.

[29] Pickett H.M. (1991). The Fitting and Prediction of Vibration-Rotation Spectra with Spin Interactions. J. Mol. Spectrosc..

[30] Gordy W., Cook R.L. (1984). Microwave Molecular Spectra.

[31] Gerhard D., Hellweg A., Merke I., Stahl W., Baudelet M., Petitprez D., Wlodarczak G. (2003). Internal Rotation and Chlorine Nuclear Quadrupole Coupling of O-Chlorotoluene Studied by Microwave Spectroscopy and Ab Initio Calculations. J. Mol. Spectrosc..

[32] Herschbach D.R. (1959). Calculation of Energy Levels for Internal Torsion and Over-All Rotation. III. J. Chem. Phys..

[33] Hartwig H., Dreizler H. (1996). The Microwave Spectrum of Trans-2,3-Eimethyloxirane in Torsional Excited States. Z. Naturforschung A.

[34] Dyke T.R., Muenter J.S. (1974). Microwave Spectrum and Structure of Hydrogen Bonded Water Dimer. J. Chem. Phys..

[35] Emamian S., Lu T., Kruse H., Emamian H. (2019). Exploring Nature and Predicting Strength of Hydrogen Bonds: A Correlation Analysis Between Atoms-in-Molecules Descriptors, Binding Energies, and Energy Components of Symmetry-Adapted Perturbation Theory. J. Comput. Chem..

[36] Boys S.F., Bernardi F. (1970). The Calculation of Small Molecular Interactions by the Differences of Separate Total Energies. Some Procedures with Reduced Errors. Mol. Phys..

[37] Lu T., Chen Q. (2023). Simple, Efficient, and Universal Energy Decomposition Analysis Method Based on Dispersion-Corrected Density Functional Theory. J. Phys. Chem. A.

[38] Iribarren I., Sánchez-Sanz G., Alkorta I., Elguero J., Trujillo C. (2021). Evaluation of Electron Density Shifts in Noncovalent Interactions. J. Phys. Chem. A.

[39] Brown G.G., Dian B.C., Douglass K.O., Geyer S.M., Shipman S.T., Pate B.H. (2008). A Broadband Fourier Transform Microwave Spectrometer Based on Chirped Pulse Excitation. Rev. Sci. Instrum..

[40] Pinacho P., Blanco S., López J.C. (2019). The Complete Conformational Panorama of Formanilide–Water Complexes: The Role of Water as a Conformational Switch. Phys. Chem. Chem. Phys..

[41] Pracht P., Bohle F., Grimme S. (2020). Automated Exploration of the Low-Energy Chemical Space with Fast Quantum Chemical Methods. Phys. Chem. Chem. Phys..

[42] Pracht P., Grimme S., Bannwarth C., Bohle F., Ehlert S., Feldmann G., Gorges J., Müller M., Neudecker T., Plett C. (2024). CREST—A Program for the Exploration of Low-Energy Molecular Chemical Space. J. Chem. Phys..

[43] Lee C., Yang W., Parr R.G. (1988). Development of the Colle-Salvetti Correlation-Energy Formula into a Functional of the Electron Density. Phys. Rev. B.

[44] Grimme S., Antony J., Ehrlich S., Krieg H. (2010). A Consistent and Accurate *Ab Initio* Parametrization of Density Functional Dispersion Correction (DFT-D) for the 94 Elements H-Pu. J. Chem. Phys..

[45] Frisch M.J., Pople J.A., Binkley J.S. (1984). Self-Consistent Molecular Orbital Methods 25. Supplementary Functions for Gaussian Basis Sets. J. Chem. Phys..

[46] Loru D., Vigorito A., Santos A.F.M., Tang J., Sanz M.E. (2019). The Axial/Equatorial Conformational Landscape and Intramolecular Dispersion: New Insights from the Rotational Spectra of Monoterpenoids. Phys. Chem. Chem. Phys..

[47] Verde A., López J.C., Blanco S. (2023). The Role of the Transient Atropisomerism and Chirality of Flurbiprofen Unveiled by Laser-Ablation Rotational Spectroscopy. Chem. A Eur. J..

[48] Møller C., Plesset M.S. (1934). Note on an Approximation Treatment for Many-Electron Systems. Phys. Rev..

[49] Frisch M.J. (2017). Gaussian 16, Revision 16.

[50] Lu T., Chen F. (2012). Multiwfn: A Multifunctional Wavefunction Analyzer. J. Comput. Chem..

[51] Johnson E.R., Keinan S., Mori-Sánchez P., Contreras-García J., Cohen A.J., Yang W. (2010). Revealing Noncovalent Interactions. J. Am. Chem. Soc..

[52] Bader R.F.W. (1991). A Quantum Theory of Molecular Structure and Its Applications. Chem. Rev..

[53] Glendening E.D., Landis C.R., Weinhold F. (2012). Natural Bond Orbital Methods. WIREs Comput. Mol. Sci..

[54] Parker T.M., Burns L.A., Parrish R.M., Ryno A.G., Sherrill C.D. (2014). Levels of Symmetry Adapted Perturbation Theory (SAPT). I. Efficiency and Performance for Interaction Energies. J. Chem. Phys..

[55] Hohenstein E.G., Sherrill C.D. (2012). Wavefunction Methods for Noncovalent Interactions. WIREs Comput. Mol. Sci..

[56] Turney J.M., Simmonett A.C., Parrish R.M., Hohenstein E.G., Evangelista F.A., Fermann J.T., Mintz B.J., Burns L.A., Wilke J.J., Abrams M.L. (2012). Psi4: An Open-source *Ab Initio* Electronic Structure Program. WIREs Comput. Mol. Sci..

