# Supplementary material for: Probing Hydrogen-Bonding Preferences and Methyl Internal Rotation in Sotolon and Sotolon-(H2O)1,2
Source: Int J Mol Sci. 2025 Jun 17;26(12):5806. doi: 10.3390/ijms26125806 (PMC12192813; doi:10.3390/ijms26125806)
Supplement: Supplementary file 1 [file ijms-26-05806-s001.zip › ijms-3694049-supplementary.pdf]

Probing Hydrogen-Bonding Preferences and Methyl Internal Rotation in Sotolon and Sotolon-(H2O)1,2

Andrés Verde, Juan Carlos López and Susana Blanco *

Sotolon is a chiral furanone derivative featuring three distinct oxygen atoms that can serve as hydrogen-bond acceptor sites, making it an ideal model system for probing water's preferential interactions with competing functional groups. In this study, the rotational spectrum of sotolon and its microsolvated complexes, representing the early stages of hydration, was investigated using chirped-pulse Fourier transform microwave (CP-FTMW) spectroscopy. The conformational landscape of sotolon is dominated by a single conformer stabilized by an intramolecular O–H···O=C hydrogen bond. Upon hydration, water molecules disrupt this interaction by forming closed hydrogen-bonded cycles, resulting in mono- and dihydrated complexes. High-level theoretical calculations underscore the central role of electrostatic interactions in stabilizing these hydrated structures. Furthermore, *A*/*E* splittings observed in the rotational spectrum, arising from the internal rotation of one of sotolon's methyl groups, provide insight into how hydration modulates the methyl internal rotation barrier.

Table S1.- Rotational parameters for sotolon conformers predicted at B3LYP-D3/6-311G++(d,p) level of theory.

Table S2.- Rotational parameters for sotolon monohydrates predicted at B3LYP-D3/6-311G++(d,p) level of theory.

Table S3.- Rotational parameters for sotolon dihydrates predicted at B3LYP-D3/6-311G++(d,p) level of theory.

Table S4.- Rotational parameters for sotolon conformers predicted at MP2/6-311G++(d,p) level of theory.

Table S5.- Rotational parameters for sotolon monohydrates predicted at MP2/6-311G++(d,p) level of theory.

Table S6.- Rotational parameters for sotolon dihydrates predicted at MP2/6-311G++(d,p) level of theory.

Table S7: Energy decomposition (kJ mol^{-1}) and percentage (%) obtained from a SAPT(0)/jun-cc-pVDZ calculation on the sotolon complexes.

Table S8. Predicted ρ, I_α,λ and F values for the two methyl groups in sotolon, sotolon-(H_2O)-Ia and sotolon-(H_2O)$_2$-Ia systems.

Table S9.- r_e structure of sotolon calculated at B3LYP-D3/6-311G++(d,p) level of theory.

Table S10.- r_e structure of sotolon-(H_2O) calculated at B3LYP-D3/6-311G++(d,p) level of theory.

Table S11.- r_e structure of sotolon-(H_2O)$_2$ calculated at B3LYP-D3/6-311G++(d,p) level of theory.

Table S12. Observed rotational frequencies and residuals (all the values in MHz) for the sotolon observed monomer *A* substate used for the SPFIT fit.

Table S13. Observed rotational frequencies and residuals (all the values in MHz) for the sotolon observed monomer *E* substate used for the SPFIT fit.

Table S14. Observed rotational frequencies and residuals (all the values in MHz) for the sotolon observed monomer *A* and *E* substates used for the XIAM fit.

Table S15. Observed rotational frequencies and residuals (all the values in MHz) for the sotolon-(H_2O) observed complex *A* substate used for the SPFIT fit.

Table S16. Observed rotational frequencies and residuals (all the values in MHz) for the sotolon-(H_2O) observed complex *E* substate used for the SPFIT fit.

Table S17. Observed rotational frequencies and residuals (all the values in MHz) for the sotolon-(H_2O) observed complex *A* and *E* substates used for the XIAM fit.

Table S18. Observed rotational frequencies and residuals (all the values in MHz) for the sotolon-(H_2O)$_2$ observed complex *A* substate used for the SPFIT fit.

Table S19. Observed rotational frequencies and residuals (all the values in MHz) for the sotolon-$(H_2O)_2$ observed complex *E* substate used for the SPFIT fit.

Table S20. Observed rotational frequencies and residuals (all the values in MHz) for the sotolon-$(H_2O)_2$ observed complex *A* and *E* substates used for the XIAM fit.

Figure S1.- Predicted conformers of sotolon. Relative energies were calculated at B3LYP-D3/6-311++G(d,p) level.

Figure S2.- Predicted conformers of sotolon-H_2O. Relative energies were calculated at B3LYP-D3/6-311++G(d,p) level.

Figure S3.- Predicted conformers of sotolon-$(H_2O)_2$. Relative energies were calculated at B3LYP-D3/6-311++G(d,p) level.

Figure S4.- Potential energy function for the rotation of the rotation of α (O_9-H_{19}-O_{18}-H_{20}) calculated at B3LYP-D3/6-311++G(d,p) level. This rotation interconverts the sotolon-(H_2O)-Ia and sotolon-(H_2O)-Ib through an interconversion barrier of 270 cm^{-1}.

Table S1.- Rotational parameters for sotolon conformers predicted at B3LYP-D3/6-311G++(d,p) level of theory.

Param. [a]	Soto-1	Soto-2
A /MHz	2229.08	2196.53
B /MHz	1679.75	1685.33
C /MHz	1023.13	1018.51
κ	0.09	0.13
P_{aa} /uÅ^2	284.0	283.0
P_{bb} /uÅ^2	209.9	213.2
P_{cc} /uÅ^2	16.8	16.9
μ_a /D	3.91	-5.95
μ_b /D	1.68	3.50
μ_c /D	-0.17	0.19
ΔE /cm^{-1}	0	2030
ΔG /cm^{-1}	0	1874

[a] A, B and C are the rotational constants. κ is the Ray asymmetry parameter κ=(2B-A-C)/(A-C). $P_{\alpha\alpha}$ (α = a, b, c) are the planar moments of inertia, derived from the inertial moments P_{cc}=(I_a+I_b-I_c)/2. μ_a, μ_b, μ_c are the electric dipole moment components along the principal inertial axes. ΔE is the electronic energy relative to the most stable conformer that has a value of -459.2829053 Hartree. ΔG is the Gibbs energy relative to the most stable conformer in terms of Gibbs energy that has a value of -459.181679 Hartree.

Table S2.- Rotational parameters for sotolon monohydrates predicted at B3LYP-D3/6-311G++(d,p) level of theory.

Param. [a]	Soto-(H_2O)-Ia	Soto-(H_2O)-Ib	Soto-(H_2O)-II	Soto-(H_2O)-III	Soto-(H_2O)-IV	Soto-(H_2O)-V
A /MHz	2104.34	2115.77	1671.28	1776.29	1654.77	1902.79
B /MHz	899.07	898.14	976.36	931.22	946.26	785.71
C /MHz	662.99	661.33	651.81	637.13	631.91	577.47
κ	-0.67	-0.67	-0.36	-0.48	-0.39	-0.69
P_{aa} /uÅ^2	542.1	544.0	495.3	525.7	514.2	626.4
P_{bb} /uÅ^2	220.2	220.2	280.1	267.5	285.5	248.8
P_{cc} /uÅ^2	20.0	18.7	22.3	17.0	19.9	16.8
μ_a /D	2.54	2.77	0.90	6.48	0.80	5.58
μ_b /D	1.28	1.08	2.43	1.91	2.80	-0.46
μ_c /D	-1.20	1.19	0.18	0.42	-1.14	0.01
ΔE /cm^{-1}	0	0.3	2131	2176	2323	2349
ΔG /cm^{-1}	0	10	1422	1312	1720	1204

[a] A, B and C are the rotational constants. κ is the Ray asymmetry parameter κ=(2B-A-C)/(A-C). $P_{\alpha\alpha}$ (α = a, b, c) are the planar moments of inertia, derived from the inertial moments P_{cc}=(I_a+I_b-I_c)/2. μ_a, μ_b, μ_c are the electric dipole moment components along the principal inertial axes. ΔE is the electronic energy relative to the most stable conformer that has a value of -535.762131 Hartree. ΔG is the Gibbs energy relative to the most stable conformer in terms of Gibbs energy that has a value of -535.639577 Hartree.

Table S3.- Rotational parameters for sotolon dihydrates predicted at B3LYP-D3/6-311G++(d,p) level of theory.

**Param. [a]**	**Soto-$(H_2O)_2$-Ia**	**Soto-$(H_2O)_2$-Ib**	**Soto-$(H_2O)_2$-Ic**	**Soto-$(H_2O)_2$-Id**
A /MHz	1678.95	1693.19	1694.66	1679.52
B /MHz	571.78	568.84	570.13	568.82
C /MHz	442.25	441.48	440.23	442.56
κ	-0.79	-0.80	-0.79	-0.80
P_{aa} /uÅ^2	862.8	867.34	868.10	864.75
P_{bb} /uÅ^2	279.9	277.38	279.89	277.19
P_{cc} /uÅ^2	21.1	21.10	18.33	23.71
μ_a /D	-2.36	-2.37	-2.33	-2.1
μ_b /D	-0.46	-0.40	-0.72	-0.9
μ_c /D	-0.01	-0.07	1.77	-1.7
ΔE /cm^{-1}	0	21	212	214
ΔG /cm^{-1}	42	32	43	0

[a] A, B and C are the rotational constants. κ is the Ray asymmetry parameter κ=(2B-A-C)/(A-C). $P_{\alpha\alpha}$ (α = a, b, c) are the planar moments of inertia, derived from the inertial moments P_{cc}=(I_a+I_b-I_c)/2. μ_a, μ_b, μ_c are the electric dipole moment components along the principal inertial axes. ΔE is the electronic energy relative to the most stable conformer. ΔG is the Gibbs energy relative to the most stable conformer in terms of Gibbs energy.

**Param. [a]**	**Soto-$(H_2O)_2$-II**	**Soto-$(H_2O)_2$-III**	**Soto-$(H_2O)_2$-IV**	**Soto-$(H_2O)_2$-V**
A /MHz	1424.16	1034.21	1366.16	1469.73
B /MHz	647.26	817.67	757.80	672.87
C /MHz	531.21	475.52	662.04	545.61
κ	-0.74	0.22	-0.73	-0.72
P_{aa} /uÅ^2	688.7	596.1	530.2	666.7
P_{bb} /uÅ^2	262.7	466.7	233.2	259.5
P_{cc} /uÅ^2	92.1	22.0	136.7	84.3
μ_a /D	-3.05	-1.14	1366.16	-2.99
μ_b /D	-4.37	-1.06	757.80	-0.62
μ_c /D	0.92	0.14	662.04	2.67
ΔE /cm^{-1}	976	1266	1333	1373
ΔG /cm^{-1}	1251	1273	1383	1447

[a] A, B and C are the rotational constants. κ is the Ray asymmetry parameter κ=(2B-A-C)/(A-C). $P_{\alpha\alpha}$ (α = a, b, c) are the planar moments of inertia, derived from the inertial moments P_{cc}=(I_a+I_b-I_c)/2. μ_a, μ_b, μ_c are the electric dipole moment components along the principal inertial axes. ΔE is the electronic energy relative to the most stable conformer. ΔG is the Gibbs energy relative to the most stable conformer in terms of Gibbs energy.

Table S4.- Rotational parameters for sotolon conformers predicted at MP2-D3/6-311G++(d,p) level of theory.

**Param.** [a]	**Sotolon**	**Sotolon2**
A /MHz	2236.43	2203.63
B /MHz	1677.70	1682.75
C /MHz	1025.72	1020.94
κ	0.08	0.12
P_{aa} /uÅ^2	284.0	283.0
P_{bb} /uÅ^2	208.7	212.0
P_{cc} /uÅ^2	17.3	17.3
μ_a /D	2.63	-5.56
μ_b /D	2.41	-2.55
μ_c /D	1.58	-2.45
ΔE /cm^{-1}	0	1820

[a] A, B and C are the rotational constants. κ is the Ray asymmetry parameter κ=(2B-A-C)/(A-C). $P_{\alpha\alpha}$ (α = a, b, c) are the planar moments of inertia, derived from the inertial moments P_{cc}=(I_a+I_b-I_c)/2. μ_a, μ_b, μ_c are the electric dipole moment components along the principal inertial axes. ΔE is the electronic energy relative to the most stable conformer that has a value of -458.0303291 Hartree.

Table S5.- Rotational parameters for sotolon monohydrates predicted at MP2/6-311G++(d,p) level of theory.

**Param.** [a]	**Soto-(H_2O)-Ia**	**Soto-(H_2O)-Ib**	**Soto-(H_2O)-II**	**Soto-(H_2O)-III**	**Soto-(H_2O)-IV**	**Soto-(H_2O)-V**
A /MHz	2108.25	2124.03	1640.03	1715.16	1639.43	1913.07
B /MHz	892.32	893.86	982.39	962.58	955.24	777.71
C /MHz	659.70	658.69	660.57	662.81	638.21	574.64
κ	-0.68	-0.68	-0.34	-0.43	-0.37	-0.70
P_{aa} /uÅ^2	546.4	547.4	485.7	496.4	506.3	632.6
P_{bb} /uÅ^2	219.7	219.9	279.4	266.1	285.5	246.9
P_{cc} /uÅ^2	20.0	18.0	28.8	28.6	22.7	17.3
μ_a /D	1.96	2.02	-0.90	-5.59	-0.07	-4.40
μ_b /D	1.08	0.99	1.93	-1.87	-2.33	-2.22
μ_c /D	-1.18	0.89	0.23	0.90	1.39	-1.62
ΔE /cm^{-1}	0	19	1678	1856	1894	2019

[a] A, B and C are the rotational constants. κ is the Ray asymmetry parameter κ=(2B-A-C)/(A-C). $P_{\alpha\alpha}$ (α = a, b, c) are the planar moments of inertia, derived from the inertial moments P_{cc}=(I_a+I_b-I_c)/2. μ_a, μ_b, μ_c are the electric dipole moment components along the principal inertial axes. ΔE is the electronic energy relative to the most stable conformer that has a value of -534.3231124 Hartree.

Table S6.- Rotational parameters for sotolon dihydrates predicted at MP2/6-311G++(d,p) level of theory.

**Param.** [a]	**Soto-$(H_2O)_2$-Ia**	**Soto-$(H_2O)_2$-Ib**	**Soto-$(H_2O)_2$-Ic**	**Soto-$(H_2O)_2$-Id**
A /MHz	1699.32	1702.54	1703.94	1647.22
B /MHz	566.31	566.52	569.44	571.61
C /MHz	439.09	439.18	441.46	451.96
κ	-0.80	-0.80	-0.80	-0.80
P_{aa} /uÅ^2	873.0	872.99	867.9	847.8
P_{bb} /uÅ^2	278.0	277.7	276.9	270.4
P_{cc} /uÅ^2	19.4	19.09	19.7	36.4
μ_a /D	-1.61	-1.62	-1.90	-1.10
μ_b /D	0.84	1.00	-0.80	1.48
μ_c /D	-0.40	-0.21	-0.72	-0.86
ΔE /cm^{-1}	0	13	168	203

[a] A, B and C are the rotational constants. κ is the Ray asymmetry parameter κ=(2B-A-C)/(A-C). $P_{\alpha\alpha}$ (α = a, b, c) are the planar moments of inertia, derived from the inertial moments P_{cc}=(I_a+I_b-I_c)/2. μ_a, μ_b, μ_c are the electric dipole moment components along the principal inertial axes. ΔE is the electronic energy relative to the most stable conformer.

**Param.** [a]	**Soto-$(H_2O)_2$-II**	**Soto-$(H_2O)_2$-III**	**Soto-$(H_2O)_2$-IV**	**Soto-$(H_2O)_2$-V**
A /MHz	1441.87	1042.61	1345.15	1455.74
B /MHz	629.87	810.67	768.69	700.21
C /MHz	518.45	479.39	687.32	585.01
κ	-0.76	0.18	-0.75	-0.74
P_{aa} /uÅ^2	713.3	596.4	508.5	619.2
P_{bb} /uÅ^2	261.5	457.8	226.8	244.6
P_{cc} /uÅ^2	89.0	27.0	148.9	102.5
μ_a /D	-2.84	-0.63	-0.41	2.02
μ_b /D	-3.91	-0.43	-0.23	1.71
μ_c /D	-0.31	-0.34	-0.03	-1.16
ΔE /cm^{-1}	1142	1381	1223	1446

[a] A, B and C are the rotational constants. κ is the Ray asymmetry parameter κ=(2B-A-C)/(A-C). $P_{\alpha\alpha}$ (α = a, b, c) are the planar moments of inertia, derived from the inertial moments P_{cc}=(I_a+I_b-I_c)/2. μ_a, μ_b, μ_c are the electric dipole moment components along the principal inertial axes. ΔE is the electronic energy relative to the most stable conformer.

Table S7: Energy decomposition (kJ mol^{-1}) and percentage (%) obtained from a SAPT(0)/jun-cc-pVDZ calculation on the sotolon complexes, E_{exch} the repulsion due to exchange, E_{ind} the induction energy accounting charge transfer interactions and E_{disp} the dispersion interactions.

SAPT	**Sotolon-(H_2O)**	**Sotolon-($H_2O)_2$**
$\Delta E_{elec}/kJmol^{-1}$	-104.8	-117.3
$\Delta E_{exch}/kJmol^{-1}$	107.7	103.5
$\Delta E_{ind}/kJmol^{-1}$	-38.6	-43.1
$\Delta E_{disp}/kJmol^{-1}$	-19.0	-20.7
$\Delta E_{tot}/kJmol^{-1}$	-54.7	-77.6
% E_{elec}	64.5	64.8
% E_{ind}	23.8	23.8
% E_{disp}	11.7	11.5

Table S8. Predicted ϱ, I_α, λ and F values for the two methyl groups in sotolon, sotolon-(H_2O)-Ia and sotolon-($H_2O)_2$-Ia detected systems.

		ρ_a	ρ_b	ρ_c	$I_\alpha/u\text{Å}^2$	F/GHz	λ_a	λ_b	λ_c
Sotolon	$C_{(8)}H_3$	0.009067	0.007974	0.000040	3.159	161.91284	0.64943	0.76039	0.00619
	$C_{(9)}H_3$	0.009137	-0.003843	-0.004260	3.170	161.09103	-0.67880	0.21876	-0.70098
Sotolon-(H_2O)	$C_{(8)}H_3$	-0.006281	-0.000041	-0.000041	3.156	161.29122	-0.47531	-0.87976	-0.00990
	$C_{(9)}H_3$	-0.009003	0.001232	-0.002906	3.168	160.88627	-0.67880	0.21876	-0.70098
Sotolon-($H_2O)_2$	$C_{(8)}H_3$	0.006091	0.002897	-0.000155	3.155	161.15234	0.58122	0.81179	-0.05631
	$C_{(9)}H_3$	0.006521	-0.001168	-0.002007	3.191	159.32155	0.61523	-0.32365	-0.71885

Table S9.- r_e structure of sotolon calculated at B3LYP-D3/6-311G++(d,p) level of theory.

**Atom**	**a**	**b**	**c**
O_1	-0.099592126	-1.484449386	-0.297971733
C_2	-1.126838729	-0.634795535	-0.056949050
C_3	-0.583040218	0.735073651	0.027748995
C_4	0.739983907	0.713883443	-0.164636591
C_5	1.138891200	-0.724952721	-0.385318520
O_6	-2.281503159	-0.961250130	0.064534178
O_7	-1.398975498	1.781802590	0.266283229
C_8	1.708171312	1.846064904	-0.176184268
C_9	2.132265849	-1.280188053	0.625477674
H_{10}	1.515191349	-0.869923442	-1.404886323
H_{11}	-2.300996127	1.435484527	0.343265714
H_{12}	3.082493582	-0.745312858	0.550625002
H_{13}	1.742025050	-1.165244505	1.639257960
H_{14}	2.313743074	-2.339126766	0.434091463
H_{15}	2.282923721	1.863509012	-1.108629286
H_{16}	2.428924117	1.757824043	0.644076231
H_{17}	1.188570644	2.799333668	-0.072838459

Table S10.- r_e structure of sotolon-(H_2O) calculated at B3LYP-D3/6-311G++(d,p) level of theory.

Atom	a	b	c
O_1	0.721582083	-1.500016848	-0.299657843
C_2	-0.468904043	-0.880308023	-0.152017046
C_3	-0.231333749	0.582647711	-0.066238048
C_4	1.088752126	0.809557255	-0.169647822
C_5	1.786742390	-0.511190778	-0.323749600
O_6	-1.516125113	-1.492414637	-0.107975178
O_7	-1.193829376	1.501724005	0.081846570
C_8	1.798713844	2.118625675	-0.144421222
C_9	2.796033139	-0.845125118	0.766013079
O_{10}	-3.585241117	0.233285381	0.307651742
H_{11}	2.256874171	-0.594365251	-1.311073764
H_{12}	2.319792436	-0.796927475	1.747843549
H_{13}	3.623270584	-0.131396964	0.740380587
H_{14}	3.197821488	-1.848592047	0.615174168
H_{15}	2.404150007	2.258262489	-1.047043099
H_{16}	2.478923332	2.184090052	0.712063479
H_{17}	1.083143981	2.938659434	-0.076704111
H_{18}	-2.091728391	1.100412277	0.161857940
H_{19}	-3.128501489	-0.616327327	0.155474219
H_{20}	-4.295714269	0.290643230	-0.337818451

Table S11.- r_e structure of sotolon-$(H_2O)_2$ calculated at B3LYP-D3/6-311G++(d,p) level of theory.

Atom	a	b	c
O_1	1.034958541	-1.517162328	-0.354206464
C_2	-0.071688191	-0.757369053	-0.197623094
C_3	0.340440786	0.659000685	-0.061486478
C_4	1.681288498	0.723253082	-0.136310915
C_5	2.214333202	-0.667623709	-0.324797138
O_6	-1.186692420	-1.231312210	-0.183813368
O_7	-0.502357463	1.677060896	0.120431113
C_8	2.546930682	1.932299903	-0.052449024
C_9	3.151025151	-1.160381729	0.769659086
O_{10}	-3.170605381	1.465400097	-0.003340516
O_{11}	-3.900012129	-1.140498113	0.279428887
H_{12}	2.691931053	-0.775335179	-1.306170303
H_{13}	4.058806489	-0.551946498	0.785704531
H_{14}	2.662028681	-1.087974672	1.743757743
H_{15}	3.431877291	-2.199601338	0.589516255
H_{16}	3.219538870	1.998879471	-0.914830589
H_{17}	3.176004880	1.909226659	0.844703984
H_{18}	1.936027323	2.835006409	-0.017558464
H_{19}	-1.460766601	1.435024490	0.049225597
H_{20}	-3.601959265	2.043663553	0.632366653
H_{21}	-3.588635300	0.581949151	0.094939977
H_{22}	-2.962573399	-1.372733334	0.148137056
H_{23}	-4.394627946	-1.650676029	-0.368257958

Table S12. Observed rotational frequencies and residuals (all the values in MHz) for the sotolon observed monomer *A* substate used for the SPFIT fit.

J′	K′-1	K′+1	J″	K″-1	K″+1	Obs.	Obs.-Cal.	J′	K′-1	K′+1	J″	K″-1	K″+1	Obs.	Obs.-Cal.
1	0	1	0	0	0	2710.2874	0.0001	3	3	1	3	1	2	5523.8965	-0.0085
4	3	1	4	2	2	2997.6709	-0.0023	4	3	2	4	1	3	5694.1771	-0.0022
4	2	2	4	2	3	3009.1221	-0.0109	4	1	3	4	1	4	5854.1235	-0.0037
1	1	1	0	0	0	3258.6350	-0.0017	4	1	3	4	0	4	5869.6825	0.0008
4	2	2	4	1	3	3415.4922	-0.0067	2	1	1	1	1	0	6077.8883	0.0018
2	2	1	2	1	2	3616.9864	0.0016	2	1	1	1	1	0	6077.8883	0.0018
3	1	2	3	1	3	3817.6515	-0.0001	4	2	3	4	1	4	6260.4965	0.0032
2	2	1	2	0	2	3845.1235	0.0043	4	2	3	4	0	4	6276.0517	0.0041
3	1	2	3	0	3	3883.4343	0.0010	3	0	3	2	1	2	6902.3224	-0.0016
3	1	2	3	0	3	3883.4344	0.0011	3	1	3	2	1	2	6968.1047	-0.0008
2	0	2	1	1	1	4535.1281	0.0000	3	0	3	2	0	2	7130.4574	-0.0010
3	2	2	3	1	3	4779.7443	0.0034	3	1	2	2	2	1	7168.7731	0.0005
3	2	2	3	0	3	4845.5264	0.0040	3	1	3	2	0	2	7196.2378	-0.0021
2	0	2	1	0	1	5083.4792	0.0015	2	2	1	1	1	0	7722.9406	0.0054
2	1	2	1	0	1	5311.6116	-0.0004	5	1	4	5	1	5	7800.8135	0.0011

Table S13. Observed rotational frequencies and residuals (all the values in MHz) for the sotolon observed monomer *E* substate used for the SPFIT fit.

J′	K′-1	K′+1	J″	K″-1	K″+1	Obs.	Obs.-Cal.	J′	K′-1	K′+1	J″	K″-1	K″+1	Obs.	Obs.-Cal.
1	0	1	0	0	0	2710.2072	-0.0024	3	3	1	3	1	2	5522.1105	-0.0013
4	3	1	4	2	2	2997.8158	-0.0065	4	3	2	4	1	3	5693.6656	0.0075
4	2	2	4	2	3	3008.8097	-0.0015	4	1	3	4	1	4	5853.5853	-0.0013
1	1	1	0	0	0	3258.5277	-0.0039	4	1	3	4	0	4	5869.1491	0.0056
4	2	2	4	1	3	3415.2118	0.0049	2	1	1	1	1	0	6077.6502	0.0034
2	2	1	2	1	2	3616.5291	-0.0032	2	1	1	1	1	0	6077.6502	0.0034
3	1	2	3	1	3	3817.2783	0.0006	4	2	3	4	1	4	6259.9920	0.0098
2	2	1	2	0	2	3844.6762	-0.0001	4	2	3	4	0	4	6275.5508	0.0117
3	1	2	3	0	3	3883.0672	0.0007	3	0	3	2	1	2	6902.2434	0.0006
3	1	2	3	0	3	3883.0672	0.0007	3	1	3	2	1	2	6968.0345	0.0028
2	0	2	1	1	1	4535.0791	0.0082	3	0	3	2	0	2	7130.3874	0.0004
3	2	2	3	1	3	4779.3560	-0.0028	3	1	2	2	2	1	7168.7731	-0.0038
3	2	2	3	0	3	4845.1483	0.0006	3	1	3	2	0	2	7196.1746	-0.0011
2	0	2	1	0	1	5083.3935	0.0006	2	2	1	1	1	0	7722.4131	-0.0035
2	1	2	1	0	1	5311.5329	-0.0040	5	1	4	5	1	5	7800.1043	-0.0200

Table S14. Observed rotational frequencies and residuals (all the values in MHz) for the sotolon observed monomer *A* and *E* substates used for the XIAM fit.

S	J′	K'_{-1}	K'_{+1}	J″	K''_{-1}	K''_{+1}	Obs.	Obs.-Cal.	J′	K'_{-1}	K'_{+1}	J″	K''_{-1}	K''_{+1}	Obs.	Obs.-Cal.	S
A	3	2	1	3	1	2	2299.5879	-0.0029	3	2	1	1	3	2	2299.5413	0.0025	*E*
A	1	0	1	0	0	0	2710.2874	0.0001	1	0	1	1	0	0	2710.2072	0.0014	*E*
A	4	3	1	4	2	2	2997.6709	-0.0055	4	3	1	1	4	2	2997.8150	-0.0019	*E*
A	4	2	2	4	2	3	3009.1221	-0.0112	4	2	2	2	4	3	3008.8090	-0.0117	*E*
A	1	1	1	0	0	0	3258.6350	-0.0026	1	1	1	1	0	0	3258.5277	0.0045	*E*
A	4	2	2	4	1	3	3415.4922	-0.0079	4	2	2	2	4	3	3415.2110	-0.0060	*E*
A	2	2	1	2	1	2	3616.9864	-0.0012	2	2	1	1	2	2	3616.5290	0.0096	*E*
A	3	1	2	3	1	3	3817.6515	-0.0016	3	1	2	2	3	3	3817.2783	0.0030	*E*
A	2	2	1	2	0	2	3845.1235	0.0011	2	2	1	1	2	2	3844.6760	0.0149	*E*
A	3	1	2	3	0	3	3883.4343	-0.0005	3	1	2	2	3	3	3883.0672	0.0028	*E*
A	3	1	2	3	0	3	3883.4344	-0.0004	3	1	2	2	3	3	3883.0672	0.0028	*E*
A	2	0	2	1	1	1	4535.1281	0.0006	2	0	2	2	1	1	4535.0791	0.0050	*E*
A	3	2	2	3	1	3	4779.7443	0.0004	3	2	2	2	3	3	4779.3560	0.0067	*E*
A	3	2	2	3	0	3	4845.5264	0.0008	3	2	2	2	3	3	4845.1483	0.0099	*E*
A	2	0	2	1	0	1	5083.4792	0.0014	2	0	2	2	1	1	5083.3935	0.0021	*E*
A	2	1	2	1	0	1	5311.6116	-0.0010	2	1	2	2	1	1	5311.5320	-0.0011	*E*
A	3	3	1	3	1	2	5523.8965	-0.0144	3	3	1	1	3	2	5522.1100	-0.0168	*E*
A	4	3	2	4	1	3	5694.1771	-0.0071	4	3	2	2	4	3	5693.6650	0.0035	*E*
A	4	1	3	4	1	4	5854.1235	-0.0064	4	1	3	3	4	4	5853.5853	0.0028	*E*
A	4	1	3	4	0	4	5869.6825	-0.0018	4	1	3	3	4	4	5869.1491	0.0014	*E*
A	2	1	1	1	1	0	6077.8883	0.0015	2	1	1	1	1	0	6077.6502	0.0033	*E*
A	2	1	1	1	1	0	6077.8883	0.0015	2	1	1	1	1	0	6077.6502	0.0033	*E*
A	4	2	3	4	1	4	6260.4965	-0.0002	4	2	3	3	4	4	6259.9920	0.0132	*E*
A	4	2	3	4	0	4	6276.0517	0.0006	4	2	3	3	4	4	6275.5500	0.0060	*E*
A	3	0	3	2	1	2	6902.3224	-0.0013	3	0	3	3	2	2	6902.2434	-0.0018	*E*
A	3	1	3	2	1	2	6968.1047	-0.0007	3	1	3	3	2	2	6968.0345	0.0002	*E*
A	3	0	3	2	0	2	7130.4574	-0.0011	3	0	3	3	2	2	7130.3874	0.0005	*E*
A	3	1	2	2	2	1	7168.7731	0.0022	3	1	2	2	2	1	7168.7731	-0.0171	*E*
A	3	1	3	2	0	2	7196.2378	-0.0024	3	1	3	3	2	2	7196.1746	-0.0014	*E*
A	2	2	1	1	1	0	7722.9406	0.0029	2	2	1	1	1	0	7722.4130	0.0129	*E*
A	5	1	4	5	1	5	7800.8135	-0.0027	5	1	4	4	5	5	7800.1043	-0.0009	*E*

Table S15. Observed rotational frequencies and residuals (all the values in MHz) for the sotolon-(H_2O) observed complex *A* substate used for the SPFIT fit.

**J′**	**K'_{-1}**	**K'_{+1}**	**J″**	**K''_{-1}**	**K''_{+1}**	**Obs.**	**Obs.-Cal.**	**J′**	**K'_{-1}**	**K'_{+1}**	**J″**	**K''_{-1}**	**K''_{+1}**	**Obs.**	**Obs.-Cal.**
1	1	1	0	0	0	2772.9558	0.0005	4	1	4	3	1	3	5685.6001	-0.0091
2	0	2	1	0	1	3070.9918	0.0066	6	3	3	6	2	4	5723.5872	0.0012
4	1	3	3	2	2	3256.6999	0.0051	5	2	4	5	1	5	5839.8955	0.0077
2	1	1	1	1	0	3332.3300	0.0025	4	0	4	3	0	3	5928.8500	-0.0031
3	2	1	3	1	2	3486.5575	-0.0004	5	2	4	5	0	5	6171.5046	0.0108
3	0	3	2	1	2	3514.0598	-0.0023	4	2	3	3	2	2	6178.3662	0.0026
6	2	4	6	1	5	3550.3736	-0.0020	4	3	2	3	3	1	6254.8104	0.0077
5	1	4	5	0	5	3737.6217	0.0008	4	3	1	3	3	0	6269.2403	0.0067
7	2	5	7	1	6	4016.5826	-0.0117	4	3	1	4	2	2	6383.4099	-0.0193
2	1	2	1	0	1	4091.9005	0.0018	4	2	2	3	2	1	6451.0326	0.0005
3	1	3	2	1	2	4286.4351	-0.0159	4	1	4	3	0	3	6457.9915	-0.0066
2	2	1	2	1	2	4362.0377	0.0061	6	2	5	6	1	6	6581.9433	-0.0002
3	0	3	2	0	2	4534.9740	-0.0017	4	1	3	3	1	2	6597.0952	-0.0021
3	2	2	2	2	1	4651.2704	0.0018	5	0	5	4	1	4	6733.9373	-0.0046
3	2	2	3	1	3	4726.8551	0.0060	4	3	2	4	2	3	6785.3747	-0.0153
3	2	1	2	2	0	4767.5634	0.0020	2	2	1	1	1	0	6999.9184	-0.0013
6	1	5	6	0	6	4862.7389	-0.0068	5	1	5	4	1	4	7065.5440	-0.0040
3	1	2	2	1	1	4978.4591	0.0009	6	3	4	6	2	5	7169.3882	-0.0064
4	0	4	3	1	3	5156.4597	-0.0045	6	1	5	5	2	4	7256.8927	-0.0041
4	2	3	4	1	4	5219.6133	0.0099	2	2	0	1	1	1	7261.2631	0.0010
5	1	4	4	2	3	5251.9532	-0.0062	5	0	5	4	0	4	7263.0921	0.0051
3	1	3	2	0	2	5307.3663	0.0017	5	1	5	4	0	4	7594.6942	0.0011
7	3	4	7	2	5	5361.0670	0.0110	5	3	3	4	3	2	7831.7540	0.0080
3	2	2	3	0	3	5499.2449	0.0070	5	3	2	4	3	1	7881.0657	0.0112

Table S16. Observed rotational frequencies and residuals (all the values in MHz) for the sotolon-(H_2O) observed complex *E* substate used for the SPFIT fit.

J'	K'_{-1}	K'_{+1}	J''	K''_{-1}	K''_{+1}	Obs.	Obs.-Cal.	J'	K'_{-1}	K'_{+1}	J''	K''_{-1}	K''_{+1}	Obs.	Obs.-Cal.
1	1	1	0	0	0	2772.8263	0.0031	3	2	2	3	0	3	5498.6065	-0.0003
4	1	3	4	0	4	2826.8536	0.0013	4	1	4	3	1	3	5685.5620	0.0086
2	1	2	1	1	1	2869.3821	0.0010	5	2	4	5	1	5	5839.4893	0.0023
2	0	2	1	0	1	3070.9162	0.0091	4	0	4	3	0	3	5928.7788	-0.0019
4	1	3	3	2	2	3257.0264	0.0003	4	2	3	3	2	2	6178.5832	0.0034
2	1	1	1	1	0	3332.1782	0.0056	4	3	2	3	3	1	6259.5196	0.0005
4	2	2	4	1	3	3340.6381	-0.0051	4	3	1	3	3	0	6264.2104	-0.0046
5	1	4	5	1	5	3405.5481	-0.0034	4	2	2	3	2	1	6450.4994	0.0010
3	2	1	3	1	2	3487.0410	0.0026	4	1	4	3	0	3	6457.9915	0.0006
3	0	3	2	1	2	3513.9764	0.0056	6	2	5	6	1	6	6581.4706	-0.0007
5	1	4	5	0	5	3737.2209	0.0020	4	1	3	3	1	2	6596.8927	-0.0008
2	1	2	1	0	1	4091.8324	0.0058	5	0	5	4	1	4	6733.8162	-0.0104
3	0	3	2	0	2	4534.8938	0.0035	6	2	5	6	0	6	6775.0944	0.0008
3	2	2	2	2	1	4653.0591	-0.0061	2	2	1	1	1	0	6997.2616	-0.0052
3	2	2	3	1	3	4726.1742	0.0048	5	1	5	4	1	4	7065.4901	-0.0038
3	2	1	2	2	0	4765.5282	0.0003	5	0	5	4	0	4	7263.0408	0.0041
3	1	2	2	1	1	4978.2974	0.0053	2	2	0	1	1	1	7263.5999	-0.0018
4	0	4	3	1	3	5156.3420	-0.0012	7	2	6	7	1	7	7433.4456	-0.0045
4	2	3	4	1	4	5219.1988	0.0030	5	1	5	4	0	4	7594.6942	-0.0098
3	1	3	2	0	2	5307.3333	0.0055								

Table S17. Observed rotational frequencies and residuals (all the values in MHz) for the sotolon-(H_2O) observed complex *A* and *E* substates used for the XIAM fit.

S	J′	K'_{-1}	K'_{+1}	J″	K''_{-1}	K''_{+1}	Obs.	Obs.-Cal.	J′	K'_{-1}	K'_{+1}	J″	K''_{-1}	K''_{+1}	Obs.	Obs.-Cal.	S
A	1	1	1	0	0	0	2772.9558	0.0018	4	1	4	3	0	3	6457.9915	-0.0057	*A*
A	2	0	2	1	0	1	3070.9918	0.0137	6	2	5	6	1	6	6581.9433	-0.0056	*A*
A	4	1	3	3	2	2	3256.6999	0.0190	4	1	3	3	1	2	6597.0952	0.0026	*A*
A	2	1	1	1	1	0	3332.3300	0.0099	5	0	5	4	1	4	6733.9373	-0.0069	*A*
A	3	2	1	3	1	2	3486.5575	-0.0089	2	2	1	1	1	0	6999.9184	-0.0027	*A*
A	3	0	3	2	1	2	3514.0598	0.0079	5	1	5	4	1	4	7065.5440	-0.0084	*A*
A	6	2	4	6	1	5	3550.3736	-0.0062	6	3	4	6	2	5	7169.3882	-0.0186	*A*
A	5	1	4	5	0	5	3737.6217	0.0020	6	1	5	5	2	4	7256.8927	-0.0128	*A*
A	7	2	5	7	1	6	4016.5825	-0.0132	2	2	0	1	1	1	7261.2631	0.0000	*A*
A	2	1	2	1	0	1	4091.9005	0.0058	5	0	5	4	0	4	7263.0921	0.0002	*A*
A	3	1	3	2	1	2	4286.4351	-0.0087	5	1	5	4	0	4	7594.6942	-0.0059	*A*
A	2	2	1	2	1	2	4362.0376	-0.0020	5	3	3	4	3	2	7831.7539	0.0049	*A*
A	3	0	3	2	0	2	4534.9740	0.0055	5	3	2	4	3	1	7881.0657	0.0085	*A*
A	3	2	2	2	2	1	4651.2703	0.0095	1	1	1	0	0	0	2772.8263	0.0078	*E*
A	3	2	2	3	1	3	4726.8551	-0.0015	4	1	3	4	0	4	2826.8535	-0.0013	*E*
A	3	2	1	2	2	0	4767.5634	0.0102	2	1	2	1	1	1	2869.3820	-0.0017	*E*
A	6	1	5	6	0	6	4862.7389	-0.0045	2	0	2	1	0	1	3070.9162	0.0043	*E*
A	3	1	2	2	1	1	4978.4591	0.0089	2	1	1	1	1	0	3332.1782	0.0014	*E*
A	4	0	4	3	1	3	5156.4597	0.0021	4	2	2	4	1	3	3340.6380	0.0148	*E*
A	4	2	3	4	1	4	5219.6133	0.0030	3	2	1	3	1	2	3487.0410	0.0217	*E*
A	5	1	4	4	2	3	5251.9531	-0.0005	3	0	3	2	1	2	3513.9764	-0.0090	*E*
A	3	1	3	2	0	2	5307.3663	0.0059	5	1	4	5	0	5	3737.2209	-0.0118	*E*
A	7	3	4	7	2	5	5361.0670	-0.0041	2	1	2	1	0	1	4091.8324	0.0109	*E*
A	3	2	2	3	0	3	5499.2449	-0.0036	3	0	3	2	0	2	4534.8938	-0.0011	*E*
A	4	1	4	3	1	3	5685.6001	-0.0052	3	2	2	2	2	1	4653.0590	-0.0108	*E*
A	6	3	3	6	2	4	5723.5872	-0.0146	3	2	2	3	1	3	4726.1742	0.0187	*E*
A	5	2	4	5	1	5	5839.8954	0.0014	3	2	1	2	2	0	4765.5282	-0.0074	*E*
A	4	0	4	3	0	3	5928.8500	0.0005	3	1	2	2	1	1	4978.2974	-0.0023	*E*
A	5	2	4	5	0	5	6171.5046	0.0024	4	0	4	3	1	3	5156.3420	-0.0154	*E*
A	4	2	3	3	2	2	6178.3662	0.0072	4	2	3	4	1	4	5219.1988	0.0143	*E*
A	4	3	2	3	3	1	6254.8104	0.0126	3	1	3	2	0	2	5307.3333	0.0117	*E*
A	4	3	1	3	3	0	6269.2403	0.0117	3	2	2	3	0	3	5498.6065	0.0244	*E*
A	4	2	2	3	2	1	6451.0326	0.0060	4	1	4	3	1	3	5685.5620	0.0043	*E*
A	4	1	4	3	0	3	6457.9915	-0.0057	5	2	4	5	1	5	5839.4893	0.0101	*E*

Table S17. Continuation.

S	J´	K´-1	K´+1	J″	K″-1	K″+1	Obs.	Obs.-Cal.	J´	K´-1	K´+1	J″	K″-1	K″+1	Obs.	Obs.-Cal.	S
E	4	0	4	3	0	3	5928.7788	-0.0053	6	2	5	6	0	6	6775.0944	0.0062	*E*
E	4	2	3	3	2	2	6178.5832	-0.0036	2	2	1	1	1	0	6997.2616	0.0070	*E*
E	4	3	2	3	3	1	6259.5196	-0.0093	5	1	5	4	1	4	7065.4901	-0.0089	*E*
E	4	3	1	3	3	0	6264.2104	-0.0116	5	0	5	4	0	4	7263.0408	0.0023	*E*
E	4	2	2	3	2	1	6450.4994	-0.0092	2	2	0	1	1	1	7263.5999	0.0129	*E*
E	4	1	4	3	0	3	6457.9915	0.0071	7	2	6	7	1	7	7433.4455	-0.0024	*E*
E	6	2	5	6	1	6	6581.4705	0.0034	5	1	5	4	0	4	7594.6942	-0.0051	*E*
E	4	1	3	3	1	2	6596.8927	-0.0120									

Table S18. Observed rotational frequencies and residuals (all the values in MHz) for the sotolon-$(H_2O)_2$ observed complex *A* substate used for the SPFIT fit.

J´	K´-1	K´+1	J″	K″-1	K″+1	Obs.	Obs.-Cal.	J´	K´-1	K´+1	J″	K″-1	K″+1	Obs.	Obs.-Cal.
3	1	3	2	1	2	2815.8410	0.0007	5	2	3	4	2	2	5184.2171	-0.0022
3	0	3	2	0	2	2971.7211	-0.0158	5	1	4	4	1	3	5286.8840	0.0049
3	2	2	2	2	1	3012.1461	0.0079	6	1	6	5	1	5	5575.5462	0.0053
3	2	1	2	2	0	3052.5521	0.0081	6	0	6	5	0	5	5722.5604	0.0007
3	1	2	2	1	1	3195.6466	0.0055	6	2	5	5	2	4	5978.5526	0.0198
4	1	4	3	1	3	3743.6725	-0.0101	6	4	3	5	4	2	6058.7123	0.0003
4	0	4	3	0	3	3917.8304	-0.0177	6	4	2	5	4	1	6059.4825	-0.0016
4	2	3	3	2	2	4008.2304	0.0013	6	3	4	5	3	3	6067.0724	-0.0031
4	3	2	3	3	1	4035.1409	0.0055	6	3	3	5	3	2	6095.1084	-0.0046
4	3	1	3	3	0	4038.2234	0.0057	6	2	4	5	2	3	6279.4716	-0.0064
4	2	2	3	2	1	4106.5481	-0.0002	6	1	5	5	1	4	6308.1307	0.0022
4	1	3	3	1	2	4247.6947	0.0056	7	1	7	6	1	6	6479.5109	0.0026
5	1	5	4	1	4	4663.7190	0.0087	7	0	7	6	0	6	6594.8147	0.0070
5	0	5	4	0	4	4833.3774	-0.0152	7	2	6	6	2	5	6949.7635	-0.0132
5	2	4	4	2	3	4997.5505	0.0000	7	4	4	6	4	3	7078.1323	-0.0018
5	3	3	4	3	2	5050.2086	0.0003	7	4	3	6	4	2	7080.6871	0.0002
5	3	2	4	3	1	5060.8994	-0.0004								

Table S19. Observed rotational frequencies and residuals (all the values in MHz) for the sotolon-$(H_2O)_2$ observed complex *E* substate used for the SPFIT fit.

J'	K'_{-1}	K'_{+1}	J''	K''_{-1}	K''_{+1}	Obs.	Obs.-Cal.	J'	K'_{-1}	K'_{+1}	J''	K''_{-1}	K''_{+1}	Obs.	Obs.-Cal.
3	1	3	2	1	2	2815.8410	-0.0063	6	3	4	5	3	3	6071.1470	0.0023
3	0	3	2	0	2	2971.7211	0.0155	6	3	3	5	3	2	6090.9018	0.0051
3	1	2	2	1	1	3195.5729	-0.0019	6	2	4	5	2	3	6279.2693	0.0021
4	1	4	3	1	3	3743.6724	0.0046	6	1	5	5	1	4	6308.0334	-0.0032
4	0	4	3	0	3	3917.8304	0.0148	7	1	7	6	1	6	6479.4540	-0.0015
4	2	3	3	2	2	4009.1756	0.0017	7	0	7	6	0	6	6594.7591	-0.0052
4	2	2	3	2	1	4105.5074	-0.0047	7	2	6	6	2	5	6949.7635	0.0044
4	1	3	3	1	2	4247.6187	-0.0013	7	4	4	6	4	3	7079.2876	-0.0013
5	1	5	4	1	4	4663.6699	-0.0130	7	4	3	6	4	2	7079.3667	-0.0037
5	0	5	4	0	4	4833.3774	0.0167	7	3	5	6	3	4	7086.1872	-0.0005
5	3	3	4	3	2	5053.8527	-0.0026	7	3	4	6	3	3	7143.1813	0.0021
5	3	2	4	3	1	5057.1323	-0.0101	7	1	6	6	1	5	7305.4553	-0.0073
5	2	3	4	2	2	5183.8521	0.0003	8	1	8	7	1	7	7376.4368	0.0064
5	1	4	4	1	3	5286.7966	-0.0010	7	2	5	6	2	4	7380.5641	0.0047
6	1	6	5	1	5	5575.4932	-0.0087	8	0	8	7	0	7	7459.9192	0.0026
6	0	6	5	0	5	5722.5197	-0.0056								

Table S20. Observed rotational frequencies and residuals (all the values in MHz) for the sotolon-(H_2O)$_2$ observed complex *A* and *E* substates used for the XIAM fit.

S	J´	K'_{-1}	K'_{+1}	J″	K''_{-1}	K''_{+1}	Obs.	Obs.-Cal.	J´	K'_{-1}	K'_{+1}	J″	K''_{-1}	K''_{+1}	Obs.	Obs.-Cal.	S
A	3	0	3	2	0	2	2971.7211	-0.0186	7	1	6	6	1	5	7305.5594	0.0003	*A*
A	3	2	2	2	2	1	3012.1460	0.0047	7	2	5	6	2	4	7380.7259	0.0021	*A*
A	3	2	1	2	2	0	3052.5520	0.0057	3	1	3	2	1	2	2815.8410	-0.0064	*E*
A	3	1	2	2	1	1	3195.6466	-0.0004	3	0	3	2	0	2	2971.7211	0.0147	*E*
A	4	1	4	3	1	3	3743.6724	-0.0069	3	1	2	2	1	1	3195.5729	-0.0016	*E*
A	4	0	4	3	0	3	3917.8303	-0.0205	4	1	4	3	1	3	3743.6724	0.0042	*E*
A	4	2	3	3	2	2	4008.2303	-0.0015	4	0	4	3	0	3	3917.8304	0.0145	*E*
A	4	3	2	3	3	1	4035.1409	0.0011	4	2	3	3	2	2	4009.1756	0.0014	*E*
A	4	3	1	3	3	0	4038.2234	0.0014	4	2	2	3	2	1	4105.5074	-0.0027	*E*
A	4	2	2	3	2	1	4106.5480	-0.0010	4	1	3	3	1	2	4247.6187	-0.0016	*E*
A	4	1	3	3	1	2	4247.6947	-0.0010	5	1	5	4	1	4	4663.6699	-0.0146	*E*
A	5	1	5	4	1	4	4663.7190	0.0155	5	0	5	4	0	4	4833.3774	0.0164	*E*
A	5	2	4	4	2	3	4997.5504	-0.0010	5	3	3	4	3	2	5053.8527	-0.0030	*E*
A	5	3	3	4	3	2	5050.2086	-0.0024	5	3	2	4	3	1	5057.1323	-0.0056	*E*
A	5	3	2	4	3	1	5060.8993	-0.0026	5	2	3	4	2	2	5183.8521	-0.0013	*E*
A	5	2	3	4	2	2	5184.2170	0.0000	5	1	4	4	1	3	5286.7966	-0.0011	*E*
A	5	1	4	4	1	3	5286.8839	-0.0013	6	1	6	5	1	5	5575.4932	-0.0130	*E*
A	6	1	6	5	1	5	5575.5462	0.0173	6	0	6	5	0	5	5722.5197	-0.0075	*E*
A	6	0	6	5	0	5	5722.5604	0.0035	6	3	4	5	3	3	6071.1470	0.0011	*E*
A	6	4	3	5	4	2	6058.7123	-0.0035	6	3	3	5	3	2	6090.9018	0.0094	*E*
A	6	4	2	5	4	1	6059.4824	-0.0054	6	1	5	5	1	4	6308.0334	-0.0011	*E*
A	6	3	4	5	3	3	6067.0723	-0.0024	7	1	7	6	1	6	6479.4540	-0.0107	*E*
A	6	3	3	5	3	2	6095.1083	-0.0021	7	0	7	6	0	6	6594.7591	-0.0110	*E*
A	6	2	4	5	2	3	6279.4716	-0.0002	7	2	6	6	2	5	6949.7635	0.0146	*E*
A	6	1	5	5	1	4	6308.1307	-0.0024	7	4	4	6	4	3	7079.2876	0.0013	*E*
A	7	1	7	6	1	6	6479.5109	0.0220	7	4	3	6	4	2	7079.3667	0.0036	*E*
A	7	0	7	6	0	6	6594.8146	0.0170	7	3	5	6	3	4	7086.1872	0.0044	*E*
A	7	2	6	6	2	5	6949.7635	-0.0052	7	3	4	6	3	3	7143.1813	-0.0026	*E*
A	7	4	4	6	4	3	7078.1323	-0.0003	7	1	6	6	1	5	7305.4553	0.0006	*E*
A	7	4	3	6	4	2	7080.6871	0.0020	8	1	8	7	1	7	7376.4368	-0.0110	*E*
A	7	3	5	6	3	4	7084.1161	-0.0017	8	0	8	7	0	7	7459.9192	-0.0099	*E*
A	7	3	4	6	3	3	7145.4270	0.0016									

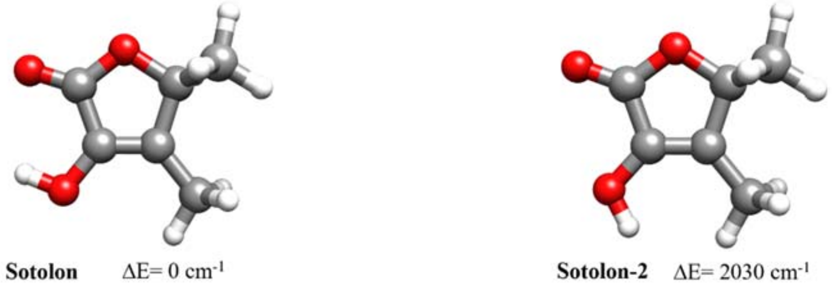

Figure S1.- Predicted conformers of sotolon. Relative energies were calculated at B3LYP-D3/6-311++G(d,p) level.

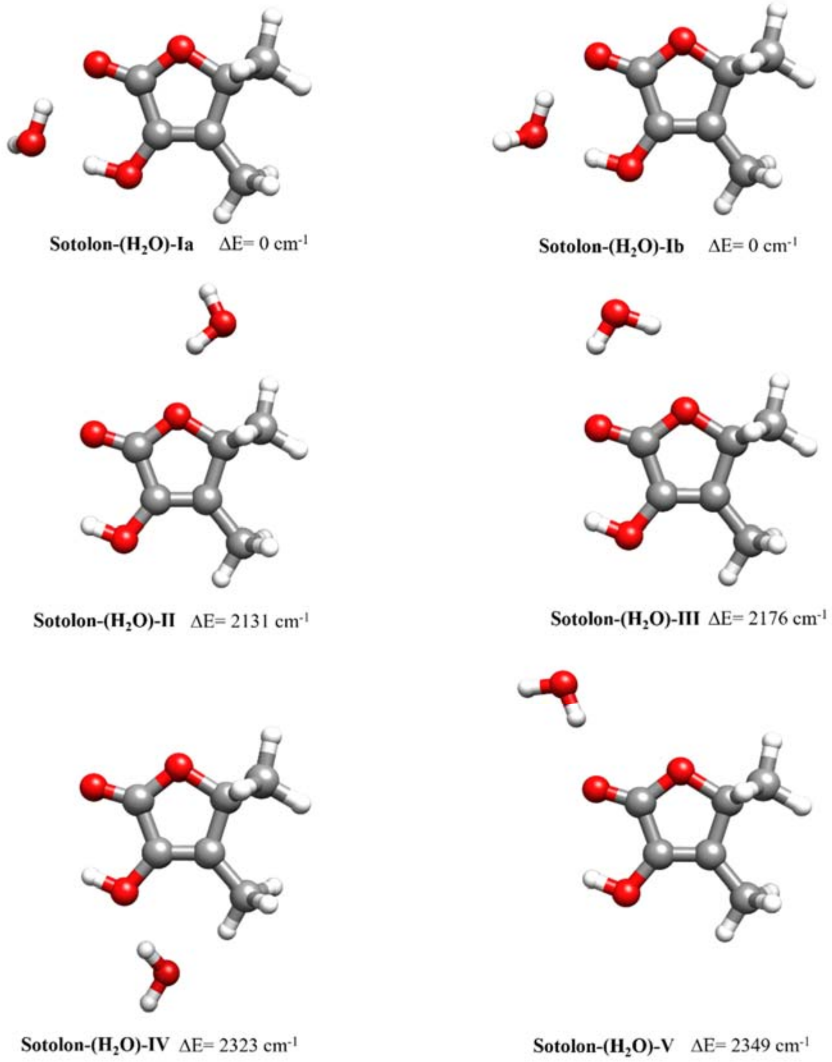

Figure S2.- Predicted conformers of sotolon-H_2O. Relative energies were calculated at B3LYP-D3/6-311++G(d,p) level.

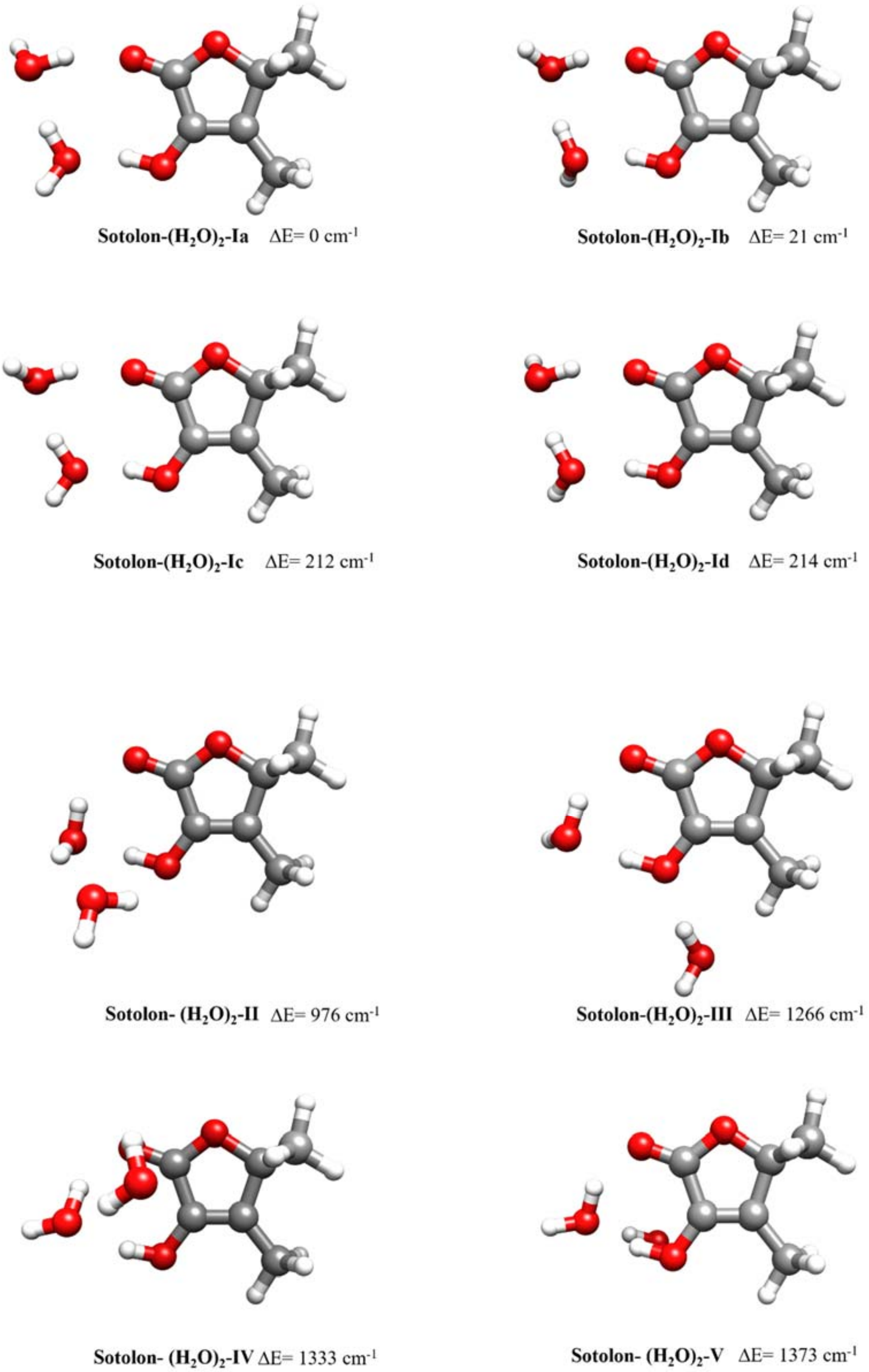

Figure S3.- Predicted conformers of sotolon-(H_2O)$_2$. Relative energies were calculated at B3LYP-D3/6-311++G(d,p) level.

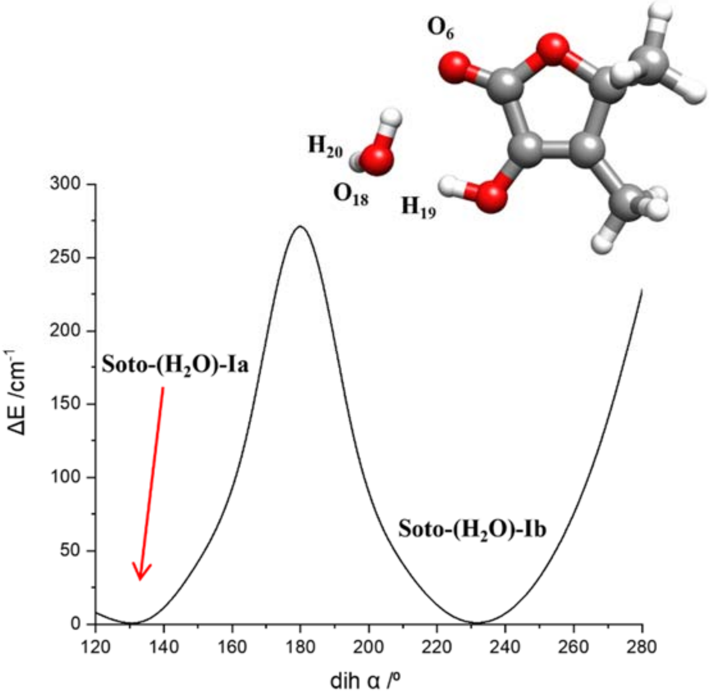

Figure S4.- Potential energy function for the rotation of the rotation of *α* (O_9-H_{19}-O_{18}-H_{20}) calculated at B3LYP-D3/6-311++G(d,p) level. This rotation interconverts the sotolon-(H_2O)-Ia and sotolon-(H_2O)-Ib through an interconversion barrier of 270 cm^{-1}.
